# Characterization of Information-Transmitting Materials Produced in Ionic Liquid-based Neuromorphic Electrochemical Devices for Physical Reservoir Computing

**DOI:** 10.1021/acsami.3c08638

**Published:** 2023-10-10

**Authors:** Dan Sato, Hisashi Shima, Takuma Matsuo, Masaharu Yonezawa, Kentaro Kinoshita, Masakazu Kobayashi, Yasuhisa Naitoh, Hiroyuki Akinaga, Shunsuke Miyamoto, Toshiki Nokami, Toshiyuki Itoh

**Affiliations:** † Device Technology Research Institute, National Institute of Advanced Industrial Science and Technology, Tsukuba, Ibaraki 305-8565, Japan; ‡ Department of Applied Physics, Graduate School of Science, Tokyo University of Science, Katsushika, Tokyo 125-8585, Japan; § New Value Creation Office, NAGASE & CO., LTD., Nihonbashi, Tokyo 103-8355, Japan; ∥ Center for Research on Green Sustainable Chemistry, Faculty of Engineering, Tottori University, Koyama, Tottori 680-8552, Japan; ⊥ Toyota Physical and Chemical Research Institute, Nagakute, Aichi 480-1192, Japan

**Keywords:** electrochemical reaction, faradaic current, ionic liquid, liquid/solid interface, reservoir computing

## Abstract

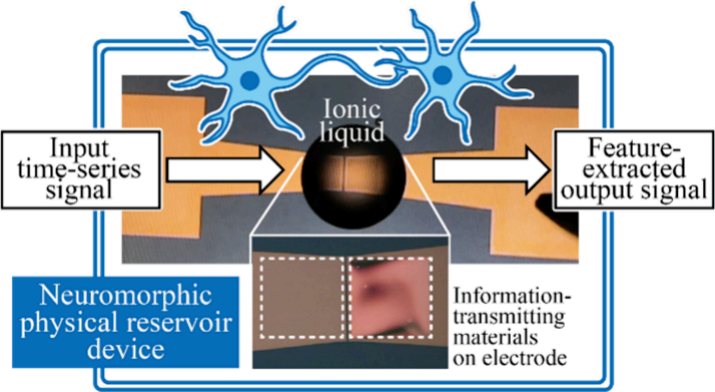

Device implementation of reservoir computing, which is expected to enable high-performance data processing in simple neural networks at a low computational cost, is an important technology to accelerate the use of artificial intelligence in the real-world edge computing domain. Here, we propose an ionic liquid-based physical reservoir device (IL-PRD), in which copper cations dissolved in an IL induce diverse electrochemical current responses. The origin of the electrochemical current from the IL-PRD was investigated spectroscopically in detail. After operating the device under various operating conditions, X-ray photoelectron spectroscopy of the IL-PRD revealed that electrochemical reactions involving Cu, Cu_2_O, Cu(OH)_2_, CuS_*x*_, and H_2_O occur at the Pt electrode/IL interface. These products are considered information transmission materials in IL-PRD similar to neurotransmitters in biological neurons. By introducing the Faradaic current components due to the electrochemical reactions of these materials into the output signal of IL-PRD, we succeeded in improving the time-series data processing performance of the nonlinear autoregressive moving average task. In addition, the information processing efficiency in machine learning to classify electrocardiogram signal waveforms was successfully improved by using the output current from IL-PRD. Optimizing the electrochemical reaction products of IL-PRD is expected to advance data processing technology in society.

## 1 Introduction

Since the human brain, which consists of a biological neural network, processes neural information extremely efficiently, neuro-inspired computing devices that physically emulate the information processing mechanisms of neurons are expected to achieve higher performance and power-saving computing.^1−3^ Physical reservoir computing (PRC) has been proposed as a hardware-based advanced computing approach, especially for processing the time-series information produced in physical space. As explained in the literature, RC is a type of recurrent neural network (RNN), but the network structure of RC is much simpler than conventional RNNs.^4−6^ More specifically, machine learning based on RC uses only three layers (input, reservoir, and output layers), pointing out some advantages of RC over other RNNs, such as complex unitary RNNs and long short-term memory networks.^7^ In addition, developing physical reservoir devices (PRDs) applicable to the reservoir layer is essential to realizing PRCs and has attracted much attention.^8−13^ We have previously proposed an ionic liquid (IL)-based physical reservoir device (IL-PRD) using imidazolium cation-based ILs with the bis(trifluoromethylsulfonyl)amide ([Tf_2_N]) anions and demonstrated efficient and accurate image classification for Modified National Institute of Standards and Technology (MNIST) database handwritten digits.^14^ In addition, the role of Cu cations in PRC of binary time-series data has been discussed using a solvated IL synthesized from equal amounts of Cu(Tf_2_N)_2_ and triglyme (G3).^15^ In the former, the charge–discharge process in the electric double layer (EDL) at the IL/metal electrode interface of the IL-PRD is the origin of the feature extraction functionality for the input data. In the latter, the electrochemical reaction of Cu cations at the IL/metal interface of the IL-PRD generates Faradaic currents, which can improve the performance of the PRC. These two information transmission mechanisms, that is, the charge–discharge process in the EDLs and electrochemical reactions, are also the fundamental steps for neural information transmission in the biological neural network. In addition, the release and uptake of neurochemical transmitters at synapses play a vital role in information transmission between adjacent neurons.^16^ In the case of IL-PRD, the products at the IL/metal electrode interface can be regarded as a kind of transmitter, because they are formed as a result of electrochemical reactions, which nonlinearly transform the input voltage signal into feature-extracted current signals. Therefore, identifying the products at the IL/metal electrode interface after the nonlinear signal transformation and the relationship between the identified products and PRC performance are informative in establishing IL-PRD development guidelines. In our previous study,^15^ although the importance of the Faradaic current generated by the electrochemical reactions involving the Cu cations in IL was confirmed, the reaction products were not determined because it was difficult to evaluate the type of the produced substances only from the measurement results of the electrical properties in IL-PRD. X-ray photoelectron spectroscopy (XPS) is often used to study the solid electrolyte interface in electrochemical devices such as batteries.^17,18^ In the present study, this technique was applied to investigate products at the IL/metal electrode interface in IL-PRD. The compound compositions on the metal electrode produced under various input voltage conditions were precisely analyzed. The accuracy of machine learning results using IL-PRD output current data was discussed, combined with the XPS results. The results indicate that in IL-PRD, the components of IL and water molecules absorbed into the IL from the atmosphere participate in the electrochemical reaction. We also found that the output current from IL-PRD improves the machine learning accuracy to classify the electrocardiogram (ECG) signal waveforms, indicating the feasibility of high-performance and power-saving computing by IL-PRD.

## 2 Experimental Section

### 2.1 IL-PRD Device Fabrication

**Figure 1:**
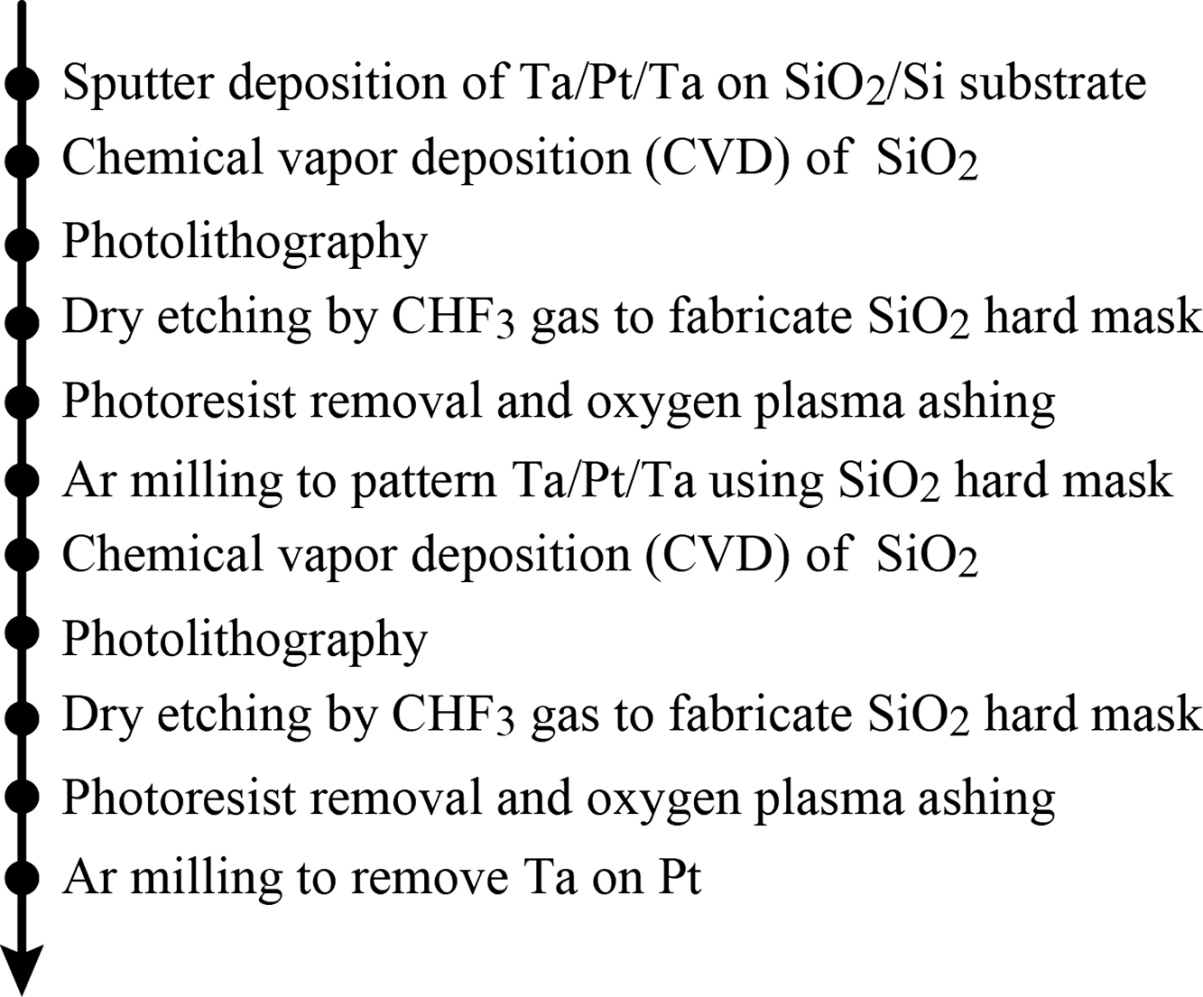
Flowchart of the IL-PRD fabrication processes.

Figure 1 shows a flowchart of the device fabrication process for the present IL-PRD. IL-PRD was fabricated on a SiO_2_/Si substrate with a SiO_2_ layer thickness of 100 nm. After sputter deposition of a Ta/Pt/Ta film stack, a CVD-SiO_2_ thin film was deposited by chemical vapor deposition (CVD). Here, the Ta layer is the adhesive. The film thicknesses were 1, 20, 1, and 30 nm for the upper Ta, Pt, lower Ta, and CVD-SiO_2_, respectively. The input and output electrodes were patterned by photolithography and dry etching processes. First, a photoresist pattern was transferred onto the CVD-SiO_2_ layer by reactive ion etching using CHF_3_ gas. Next, after removal of the photoresist on the CVD-SiO_2_, the input and output electrodes of the Ta/Pt/Ta film stack were patterned by the Ar ion milling process using the CVD-SiO_2_ pattern as a hard mask. Finally, a second CVD process was performed to cover the sidewalls of the electrode patterns, and photolithography was used to form a square Pt-exposed region where the electrochemical reaction occurs defining the IL/metal electrode interface. Note that inside the square-shaped region, not only the CVD-SiO_2_ layer but also the Ta layer above Pt is completely removed to expose Pt. The region of the input and output electrodes where Pt is exposed is hereinafter referred to as the “reaction sites”.

**Figure 2:**
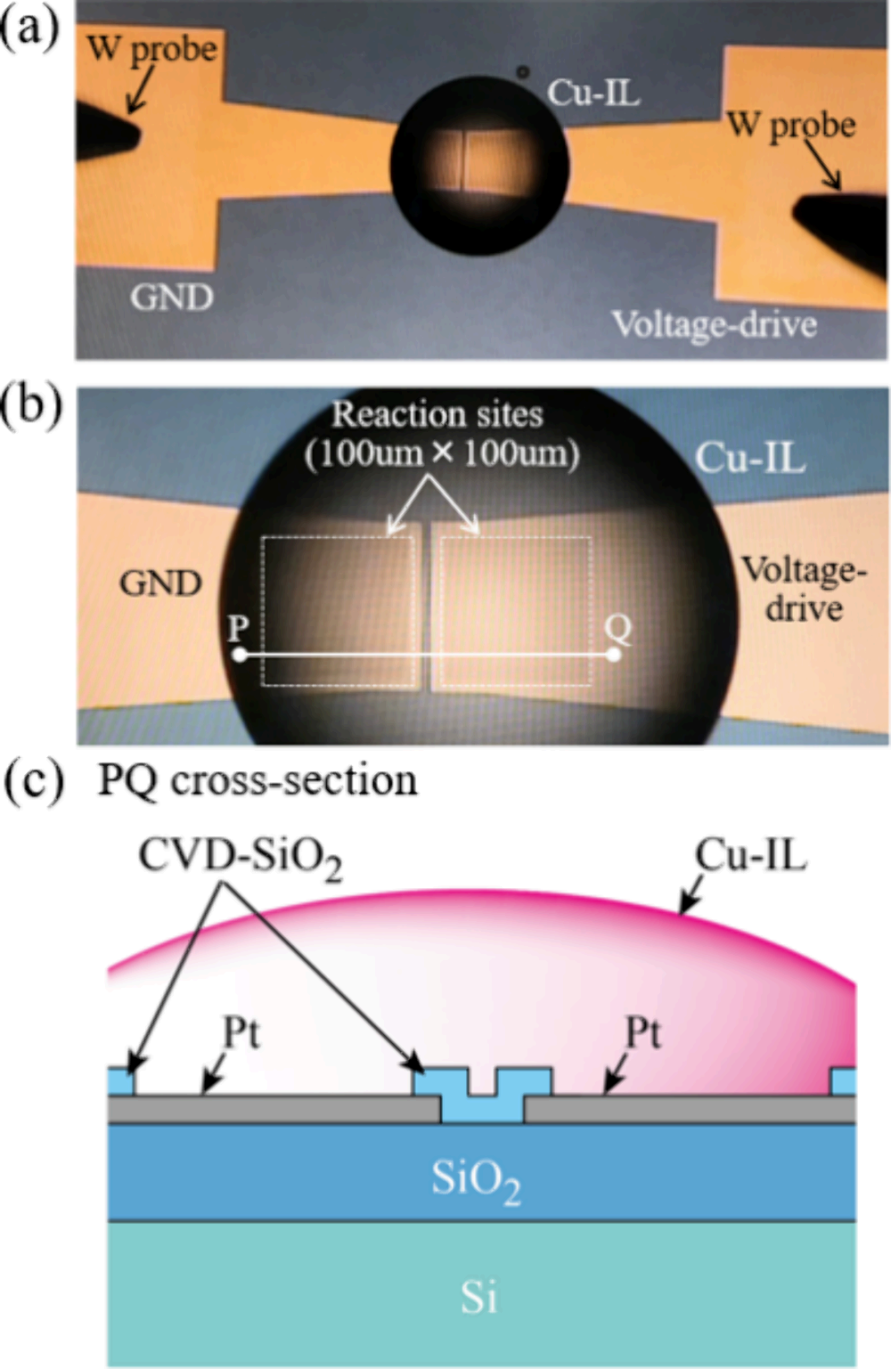
Optical microscopic images of (a) IL-PRD, (b) reaction sites with a size of 100 μm × 100 μm, and (c) schematic of the PQ cross-section in (b). The surface material of the reaction sites is Pt.

The reaction site was 100 × 100 μm in size for electrical measurements and 300 × 300 μm for XPS measurements. Prior to the electrical measurements, microdroplets of IL were adhered to the tip of a tungsten needle and placed on the reaction site to form IL/Pt electrode contacts at the reaction site. Details of the IL droplet method are described elsewhere.^19^ In this study, we used an IL containing Cu^2+^. More specifically, 1-butyl-3-methylimidazolium bis(trifluoromethylsulfonyl)amide ([BMIM][Tf_2_N]), which contains 0.4 M Cu(Tf_2_N)_2_ was used. For simplicity, this IL is denoted as “Cu-IL”. Figure 2a,b shows photographs of the IL-PRD for electrical measurements after placement of the Cu-IL droplets. A schematic cross-section of the reaction site is also shown in Figure 2c.

### 2.2 Characterization of Cu-IL

Fourier transform infrared spectroscopy (FT-IR: JASCO Corporation, FTIR-6600FV) was used to characterize Cu-IL. FT-IR measurements by the reflection method were conducted at room temperature and in air. Cu-IL droplets on the Pt/Ta/SiO_2_/Si substrate were measured. The thicknesses of Pt, Ta, and SiO_2_ films were the same as those used in the device fabrication. The wavenumber resolution was 4 cm^–1^. The FT-IR spectrum of pure [BMIM][Tf_2_N] was also measured for comparison. We also measured the FT-IR spectrum of water because the water content in IL was reportedly increased by adding a meal salt such as Cu(Tf_2_N)_2_.^20^

### 2.3 Electrical Measurements

A triangular-waveform voltage pulse (TVP) was applied to the IL-PRD as an input signal to obtain the output current signal from the IL-PRD. The voltage pulse width (PW) was fixed at 500 ms, and the voltage pulse height (PH) was varied from 1.0 to 2.5 V. Pulse voltage application and current readout were conducted by using a Keysight B1530A waveform generator/fast measurement unit mounted on a Keysight B1500 semiconductor parameter analyzer. Since sample preparation for XPS requires a relatively slow voltage sweep rate to stop the voltage application at the exact moment when the current value changes, the direct-current (DC) voltage measurement function of the source measure unit mounted on the B1500 was used. Electrical measurements were performed under a normal atmosphere (no humidity control). Humidity was approximately 10–15% or 40–50%. Electrical measurements were also performed in a dry synthetic air atmosphere for comparison. A photograph of the prober system (with gas exhaust and gas introduction functions) used in this experiment is provided in Figure S1.

### 2.4 XPS Measurements

Before XPS measurements, Cu-IL droplets were washed with acetone, and the samples were immediately transferred to a desiccator whose internal gas was purged by a high-purity (99.99%) nitrogen gas (see Figure S2). The pressure of the nitrogen gas was higher than the atmosphere pressure. After that, the samples were introduced into the XPS measurement system (ULVAC-PHI Quantera II) without exposure to the air. Then, XPS measurements were performed by using an Al Kα monochromatic source (photon energy = 1486.6 eV). The detection angle was 45°, corresponding to a detection depth of about 4–5 nm. The detection area was approximately 100 μm in diameter, sufficiently smaller than the reaction site area (300 μm × 300 μm). Therefore, XPS spectra of the reaction sites on the input and output electrodes can be obtained independently as shown in Figure S3. The Cu 2*p* 3/2, N 1*s*, O 1*s*, C 1*s*, and S 2*p* XPS spectra and the Cu LMM Auger spectra were analyzed in detail using samples prepared under different input voltage conditions. In these XPS measurements, surface cleaning by Ar ion bombardment was not conducted to prevent element-selective etching. As a control experiment, XPS measurement was performed on a sample prepared without applying external voltage after Cu-IL droplets were placed on the reaction site.

### 2.5 Machine Learning for Time-series Data

To evaluate the processing performance of IL-PRD for the time-series data, we used two kinds of benchmark tasks. One is a short-term memory (STM) task. As explained in detail in the literature and references therein,^15^ a randomly selected sequence of binary data (0 and 1), denoted as *u*(*T*), was input to IL-PRD. The target data *y*(*T*) at the time step *T* in the STM task is defined as follows:
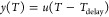
1where *T*_delay_ is a delay time. In this STM task, the amount of memory of the input signal for the past *T*_delay_ time steps can be evaluated.

The other is a nonlinear autoregressive moving average (NARMA) task with second-order dynamics, that is, NARMA2. The target data *y*(*T*) at the time step *T* ≥ 2 in NARMA2 are represented as follows:^21,22^
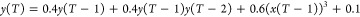
2

At *T* < 2, *y*(0) and *y*(1) were both defined as 0. Here, *x*(*T*) is originally an independent uniform noise in the interval [0, 0.5]. In this study, *x*(*T*) is generated from *u*(*T*). The maximum value of *T* is set to 200. The relationship between *x*(*T*) and *u*(*T*) is *x*(*T*) = 0.5 *u*(*T*). The STM and NARMA2 task evaluation processes for IL-PRD from input voltage application to linear regression analysis with output current values are summarized in Figure S4.

As an index of calculation performance for the STM task, the memory capacity (MC) determined below was evaluated as:
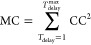
3where CC is a correlation coefficient between *y*(*T*) and model output *y*_M_(*T*). *T*_delay_^max^ is the maximum value of *T*_delay_. In the present study, *T*_delay_^max^ = 3.

On the other hand, as an index of calculation performance for the NARMA2 task, two items were evaluated using *y*_M_(*T*). One is the value of CC between *y*(*T*) and *y*_M_(*T*). The other is the normalized mean square error (NMSE), which is defined as follows:
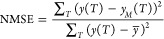
4

where *y̅* is a time average of *y*(*T*). As a control experiment, an NARMA2 task evaluation was also conducted by using a discrete resistor with a resistance value of 2 kΩ as a physical reservoir.

### 2.6 Machine Learning for ECG Signals

In order to demonstrate the feasibility of efficient machine learning, especially for the vital data by using IL-PRD, the ECG signal waveform classification task was carried out, and the classification accuracy was evaluated. In the present study, three types of ECG signals, that is, arrhythmia (ARR), congestive heart failure (CHF), and normal sinus rhythm (NSR) states, were used as classes in a classification machine learning. For each of 3 classes, 10 signals having a slightly different waveform, which are labeled as ARR_1 to ARR_10, CHF_1 to CHF_10, and NSR_1 to NSR_10, were input to IL-PRD as voltage pulses (see Figure S5). The value of PH was adjusted based on the operating voltage of IL-PRD. The value of the PW was fixed to be 500 ms. The original data of the present ECG signal waveform were downloaded from the repository.^23−25^ We measured the output current values from IL-PRD, which were used as a predictor variable (feature amount) to be processed in the output layer of PRC. In analogy with the STM and NARMA2 tasks, the virtual node technique was applied. The virtual node number *k* was set to 5 in order to downscale the output layer of PRC. For processing the above-mentioned output current from IL-PRD mapped to the five-dimensional space by the virtual nodes, a neural network consisting of input, fully connected, activation (Softmax function), and output layers were used to conduct the three-class classification in the output layer of PRC. The stochastic gradient descent algorithm was used to optimize the weight values of the neural network. The performance of the present ECG signal waveform classification was quantified based on the confusion matrix. A total of 600 output current waveforms were acquired by applying 200 each of ARR, CHF, and NSR voltage pulses. In the training phase of the neural network, 300 output current waveforms corresponding to 100 each of ARR, CHF, and NSR voltage pulses were used. On the other hand, in the prediction phase, the remaining 300 output current waveforms were used. Therefore, the classification accuracy was calculated by dividing the total number of correct predictions by 300. The learning and prediction processes were repeated 10 times, and the average value of the classification accuracy was evaluated.

## 3 Results and Discussion

### 3.1 Material Characterization of Cu-IL by FT-IR

**Figure 3:**
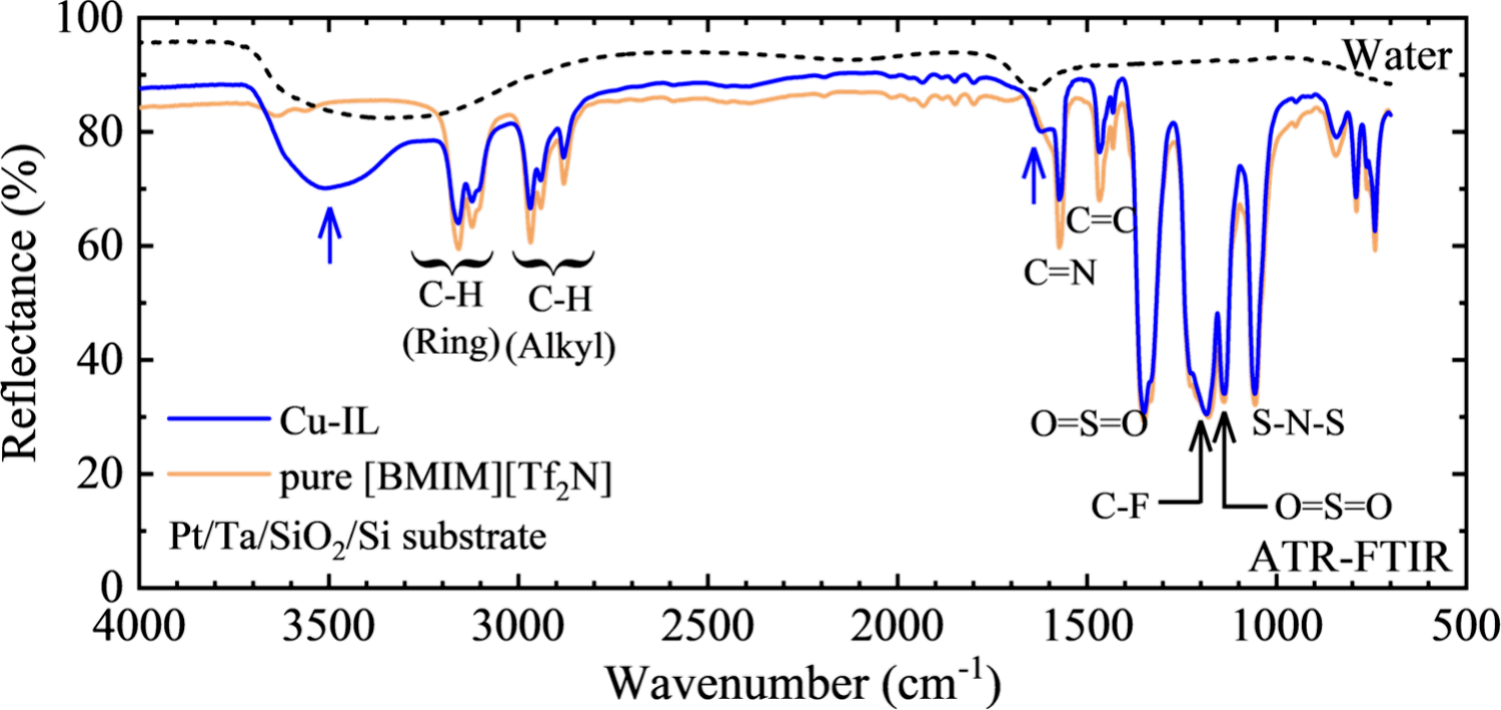
FT-IR spectra for Cu-IL (blue) and pure [BMIM][Tf_2_N] (orange) on the Pt/Ta/SiO_2_/Si substrate as well as that for water (black dotted line) measured in this study as a reference. The assignment of some typical vibrations was conducted based on the previous data, which were reproduced from ref (26) with permission from the Royal Society of Chemistry^26^ and from ref (27) with permission from Elsevier, License Number 5615270621375.^27^ The blue arrows correspond to absorption by water, because they were also found in the FT-IR spectrum for water measured in the present study.

Figure 3 shows the FT-IR measurements of Cu-IL and pure [BMIM][Tf_2_N] on a Pt/Ta/SiO_2_/Si substrate as well as water. The FT-IR spectra of pure [BMIM][Tf_2_N] were almost the same as those previously reported.^26,27^ The effect of the addition of Cu cations on the FT-IR spectra is mainly confirmed by the decrease in the absorption of vibrations such as C=C (1460 cm^–1^), C=N (1570 cm^–1^), and C–H (2800∼3200 cm^–1^) bonding in [BMIM]^+^. In contrast, the absorption by [Tf_2_N]^−^, such as S–N–S (1050 cm^–1^), O=S=O (1140 and 1350 cm^–1^), and C–F (1180 cm^–1^) bonding in Cu-IL, is almost identical to that observed in pure [BMIM][Tf_2_N]. On the other hand, the FT-IR spectra of Cu-IL and pure [BMIM][Tf_2_N] show large differences at about 1620 and 3500 cm^–2^, respectively (indicated by blue arrows). When compared with the FT-IR spectrum of water measured in the present study (black dotted line in Figure 3), these absorptions are attributed to water molecules, which is also consistent with the previous report.^28^ Therefore, the present FT-IR results suggest that Cu-IL contains a certain amount of water. Although [BMIM][Tf_2_N] is hydrophobic, the addition of Cu(Tf_2_N)_2_ increases the water absorption capacity of [BMIM][Tf_2_N]. Furthermore, it is difficult to decrease the water content in Cu-bearing [BMIM][Tf_2_N] even by overnight drying under a vacuum.^29^ Therefore, the FT-IR results may reflect the intrinsic material properties of Cu-IL. The increase of water content by adding Cu(Tf_2_N)_2_ probably originated from the coordination of water molecules around the Cu ions.^20^ It should be noted that the baselines of the FT-IR spectra between Cu-IL and pure [BMIM][Tf_2_N] were slightly different, which is thought to be caused by the difference in the IL droplet shape such as the thickness of the IL droplet.

### 3.2 Current–Voltage Characteristics of IL-PRD Using Cu-IL

**Figure 4:**
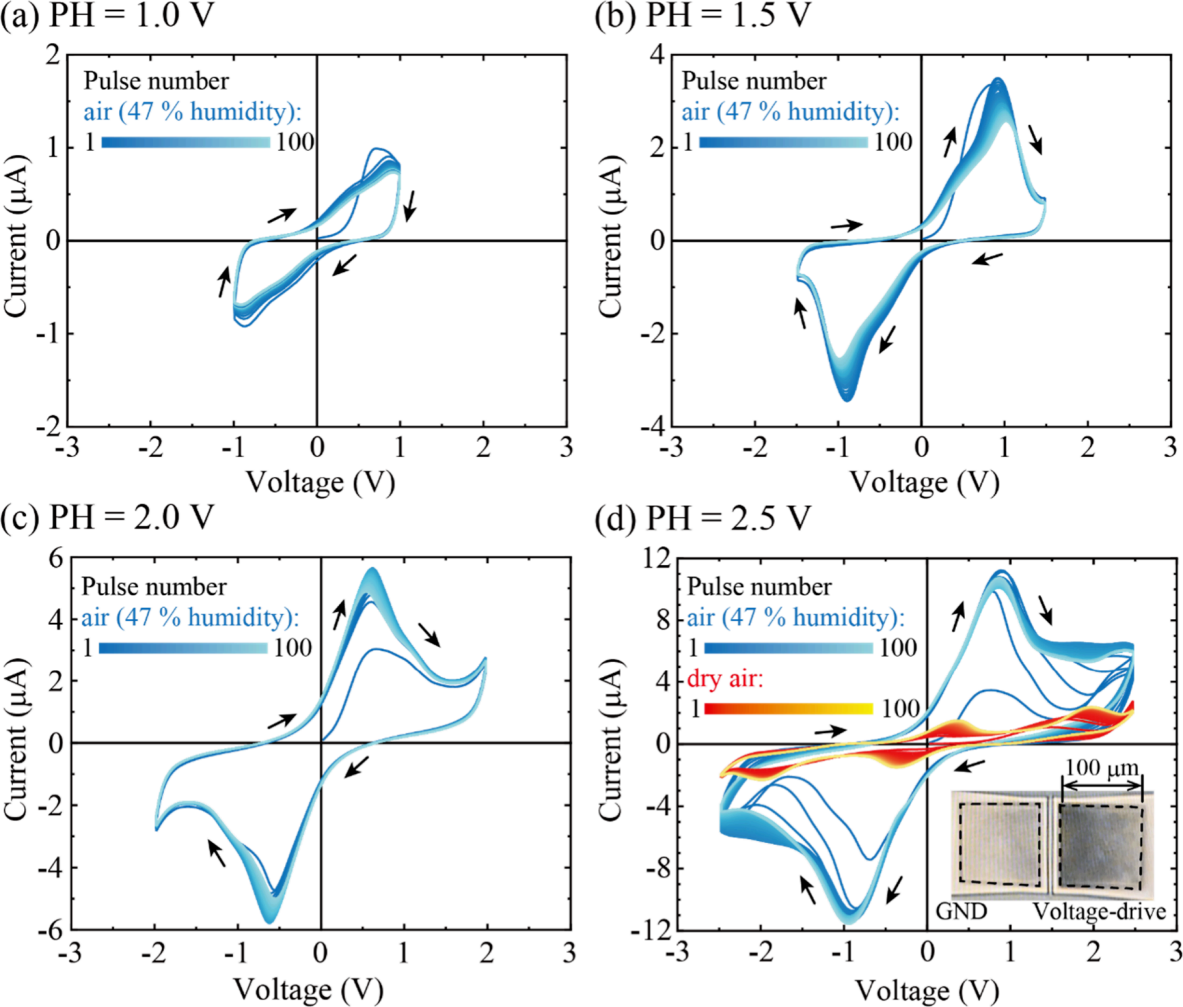
*I*-*V* curves of IL-PRD measured using triangular voltage pulses (TVPs) with different pulse heights (PH) in air with 47% humidity (blue). PH values are (a) 1.0 V, (b) 1.5 V, (c) 2.0 V, and (d) 2.5 V. The pulse width is fixed at 500 ms, and a total of 100 TVPs are applied. Positive and negative TVPs are applied alternatively. The arrows in the images are the direction of the voltage sweep. The *I*-*V* curve of IL-PRD measured in synthetic dry air is also plotted in (d) (orange). The inset in (d) is a photograph of the reaction sites showing the change in appearance due to reaction products. The reaction sites are inside the dotted squares in the inset in (d).

Figure 4a–d shows the current response of IL-PRD in a normal atmosphere as a function of the applied voltage value when positive and negative TVPs have been applied alternatively (solid blue lines). Here, PH was varied from 1.0 to 2.5 V, and PW was fixed at 500 ms. A total of 100 TVPs were applied for each PH. At PH = 1.0 V, an almost elliptical and featureless hysteresis was observed. More complex hysteresis behavior was observed as the maximum current increased with increasing PH and the current peaks became more pronounced. More specifically, when we focus on the current peak under the positive voltage, the peak current value for PH = 1.5, 2.0, and 2.5 V was approximately 3.5, 5.5, and 11 μA. For PH = 2.0 and 2.5 V (Figure 4c,d), the current peak intensity increased with the increasing number of TVPs, and finally, a stable current waveform was observed. This current peak was clearly observed at least up to 5000 cycles of the TVP application (i.e., up to 10,000th TVP), which is shown in Figure S6.

The origin of these current peaks is considered to be Faradaic currents due to redox reactions at the reaction sites. The inset of Figure 4d is a photograph showing the appearance (color) change of the reaction site (inside the black dotted squares), indicating that some reaction products are formed at the Cu-IL/Pt electrode interface. As shown in Figure 4d, the current values in dry synthetic air plotted by the solid orange lines are relatively smaller than those in normal air. This indicates that water molecules in the atmosphere around the IL-PRD are involved in the electrochemical reactions at the Cu-IL/Pt electrode interface. Then, the products formed at the reaction site were evaluated by XPS.

### 3.3 XPS Analysis of Reaction Products at the IL/Pt Electrode Interface

Five devices (10 reaction sites since one IL-PRD has 2 reaction sites at the input and output electrodes) were fabricated for XPS analysis under different applied voltage conditions. Each reaction site was named for easy distinction and is summarized in Table 1 along with the applied voltage conditions.

**Table 1:** Summary of Reaction Site Names and Voltage Sweep Sequence Conditions for XPS Analysis^a^

device ID for XPS analysis	reaction site ID on grounded electrode (output)	reaction site ID on voltage-driven electrode (input)	voltage sweep sequence
X1	1A	1B	0 → +3.0 V
X2	2A	2B	0 → +3.0 → 0 → −0.5 V
X3	3A	3B	0 → +3.0 → 0 → −1.5 V
X4	4A	4B	0 → +3.0 → 0 → −3.0
X5	5A	5B	3 cycles of 0 → +3.0 → 0 → −3.0 → 0 V

a A DC voltage sweep was used instead of a triangular voltage pulse in order to precisely adjust the final voltage value in the row of the voltage sweep sequence according to the shape of the current–voltage curve. An integration time of 200 ms/measurement point was set for this measurement. The voltage increment for each measurement step was set at 20 mV. The reaction site area is 300 μm × 300 μm.

**Figure 5:**
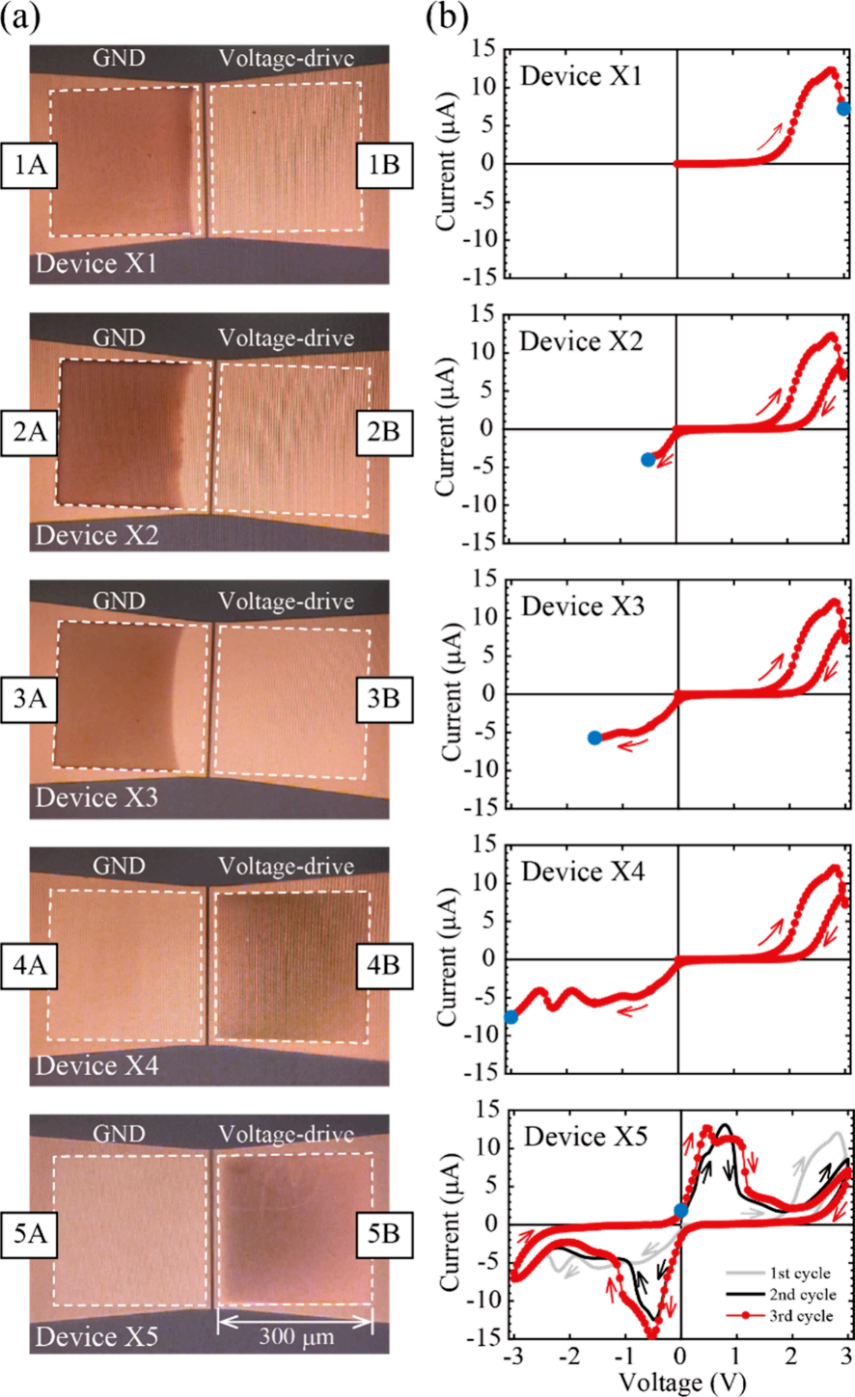
(a) Optical microscopic images of the reaction sites of the samples for XPS prepared by applying various external voltage conditions and (b) corresponding *I*-*V* curves measured during XPS sample preparation. In (a), for the sake of clarity, the edges of the reaction sites are surrounded by white dotted lines. The reaction site ID numbers are also shown in Table 1 (1A to 5A for ground electrodes and 1B to 5B for voltage-driven electrodes). The solid blue circles in (b) are the values of the last applied voltage. The arrows in (b) indicate the sweeping direction of the voltage.

**Figure 6:**
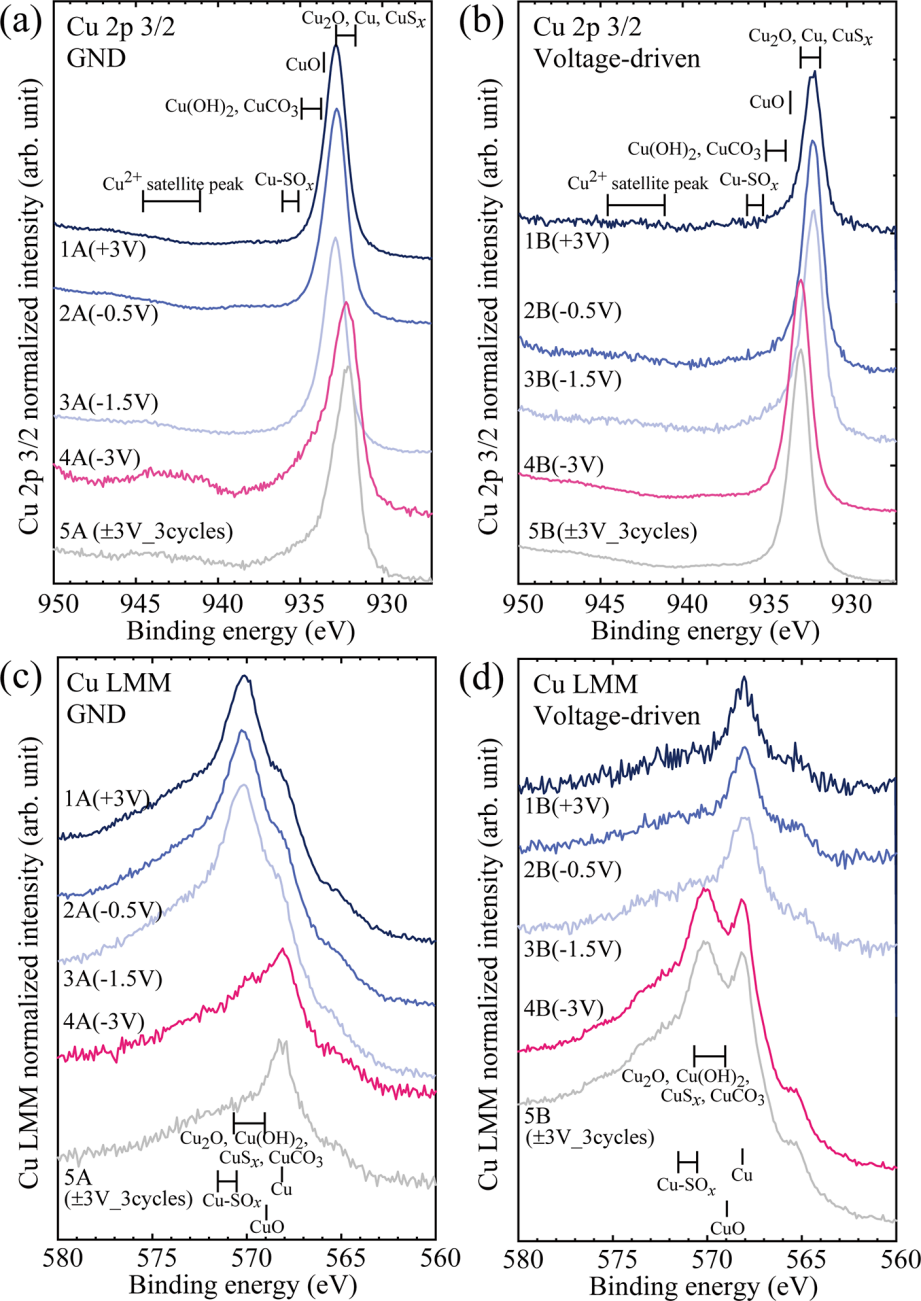
(a, b) Cu 2*p* 3/2 XPS and (c, d) Cu LMM Auger spectra of the reaction sites at the ground electrodes (a, c) and voltage-driven electrodes (b, d) measured by applying various external voltages. Cu-IL droplets were rinsed before the XPS measurements. The reaction site ID numbers in Table 1 (1A to 5A for the ground electrodes and 1B to 5B for voltage-driven electrodes) are also shown in (a) through (d).

Figure 5a shows optical micrographs of the 10 reaction sites analyzed. The current–voltage characteristics of the samples prepared for XPS analysis are shown in Figure 5b. Compared to the reaction site area of 300 μm × 300 μm, the detection area with a set value of 100 μm diameter is smaller. However, considering the geometric relationship between the X-ray source, sample surface, and photoelectron analyzer, the net detection area becomes close to the reaction site area. The estimated net detection area in the present XPS is shown in Figure S3 in the Supporting Information. Figure 6 shows the Cu 2*p* 3/2 XPS (Figure 6a,b) and Cu LMM Auger (Figure 6c,d) spectra for the eight reaction sites. The spectra in Figure 6a,c were obtained from reaction sites (1A, 2A, 3A, 4A, and 5A) on the ground electrode. The spectra in Figure 6b,d were obtained from reaction sites (1B, 2B, 3B, 4B, and 5B) on the voltage-driven electrode. Reference peak positions for metallic Cu and some Cu compounds are also shown in these figures.^30^ XPS spectra for C 1*s*, N 1*s*, O 1*s*, and S 2*p* are provided in Figures S7–S10. In these XPS measurements, F was not detected. Figure 6 shows that Cu metal and some Cu compounds (Cu_2_O, Cu(OH)_2_, and CuS_*x*_) are formed and that their abundance ratio depends on the applied voltage. Surprisingly, comparing the Cu 2*p* 3/2 XPS signals from reaction sites 1A and 1B, they were detected not only from 1A but also from 1B, despite applying the positive voltage of +3.0 V to 1B. The Cu 2*p* 3/2 XPS signal in 1A is very reasonable because when a positive voltage of +3.0 V is applied to 1B, the Cu cation having a positive charge moves toward 1A. The reduction reaction of Cu-IL from Cu cations to metallic Cu on 1A requires a reverse oxidization reaction on 1B. This can be due to the decomposition of [Tf_2_N]^−^ in Cu-IL and/or water molecules. According to the results of the control experiment (see Figure S11 for details), the Cu 2*p* 3/2 signal was very small when no external voltage was applied after the Cu-IL droplets were placed on the reaction site. Therefore, the Cu 2*p* 3/2 XPS signal detected in 1B cannot originate from the Cu-IL residues. A possible cause of the Cu 2*p* 3/2 signal detected in 1B is the reverse reaction that occurs when the external voltage is removed. Such reverse reactions are often observed in electrochemical capacitors.^31^ In the present case, reaction site 1B under an external voltage of +3.0 V is where the oxidation reaction must proceed when the voltage is maintained at +3.0 V. However, when the external voltage is removed, reduction reactions occur with metallic Cu deposition in 1B, while oxidation reactions occur with Cu_2_O formation in 1A. In addition to the deposition of metallic Cu, the valence states of the Cu cations in the Cu compounds formed at the reaction sites change with an external voltage. As shown in Figure 6a, the peak signal of the Cu^2+^ satellite is negligibly low at ground electrodes 1A, 2A, and 3A, suggesting that Cu^+^ is more dominant than Cu^2+^. With the increasing external voltage in the negative direction (4A and 5A), the peak signal of the Cu^2+^ satellite from the reaction site increases. At the same time, the main peak position in the Cu 2*p* 3/2 XPS spectra at electrode 4A shifted toward lower binding energy. On the other hand, Figure 6b shows that the peak shift toward the higher binding energy was observed at electrode 4B. The Cu^2+^ satellite peak signal from the reaction site decreases as the external voltage in the negative direction increases from −0.5 V (2B) to −3.0 V (5B). The existence of metallic Cu is confirmed from the Cu LMM Auger spectra shown in Figure 6c,d. However, the peak positions of metallic Cu and Cu^+^ are indistinguishable from the Cu 2*p* 3/2 spectra shown in Figure 6a,b. The shapes of the Cu 2*p* XPS and Cu LMM Auger spectra vary with the external voltage conditions. Therefore, it is reasonable to assume that the obtained results originate from the applied voltage and not from the contact between the reaction products and air.

Cu(OH)_2_ and CuCO_3_ are difficult to distinguish by Cu 2*p* and Cu LMM spectra because their peak positions are close in the Cu 2*p* and O 1*s* XPS spectra and Cu LMM Auger spectra. However, judging from the C 1*s* XPS spectra (see Figure S7), Cu(OH)_2_ is considered to be dominant because the peak structure at binding energies between 289 and 290 eV, which is a typical sign of CuCO_3_, was not observed.^32^ The S 2*p* XPS spectra (see Figure S10) show that S was detected at both reaction sites, although the signal intensity was relatively small, indicating that [Tf_2_N]^−^ contributed to the electrochemical reactions at the reaction sites. The concentration of detected S was approximately 2∼4 at. %, which is sufficiently larger than the detection limit (1 at. %) in the present XPS measurement. The S signal detected in the XPS spectrum presented here may be related to the decomposition of [Tf_2_N]^−^. In previous studies of IL battery electrolytes consisting of Li(Tf_2_N)/Py_1,3_(Tf_2_N), where Py_1,3_(Tf_2_N) is 1-methyl-1-propylpyrrolidinium bis(trifluoromethylsulfonyl)amide, the decomposition of [Tf_2_N]^−^ is observed during battery operation.^33^ In this literature, it is noted that the decomposition of [Tf_2_N]^−^ produces S-containing fragment species such as an anionic radical SO_2_^–·^, which can react with Li to form various Li sulfates.^33^ In the present study, S is thought to be in the CuS_*x*_ state rather than CuSO_*x*_ because a clear S 2*p* signal peak corresponding to a metal-S bonding was observed (see Figure S10).

**Figure 7:**
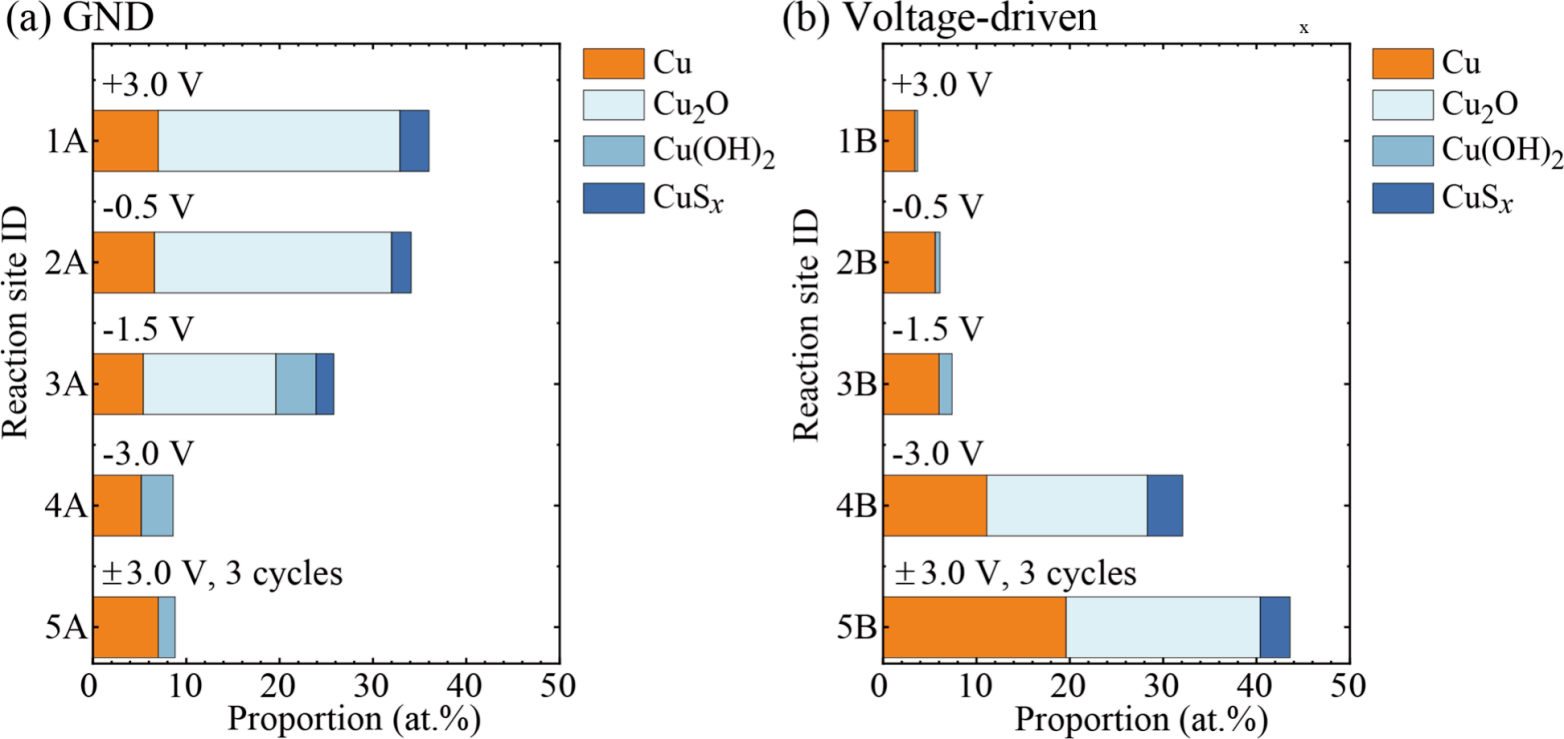
Proportion of metallic Cu and Cu compounds (Cu_2_O, Cu(OH)_2_, and CuS_*x*_) at the reaction sites on (a) ground electrodes and (b) voltage-driven electrodes. The vertical axes in (a) and (b) are the reaction site ID numbers in Table 1 (1A to 5A for ground electrodes and 1B to 5B for voltage-driven electrodes).

Careful analysis of the Cu 2*p* 3/2 XPS and Cu LMM Auger spectra reveals the proportion of Cu compounds at each reaction site in Figure 5a. In the present analysis, the values of the percentage for Cu^2+^ and the sum of Cu^0^ and Cu^+^ were calculated from the waveform separation analysis results for Cu 2*p* XPS spectra. Also, the ratio between Cu^0^ and Cu^+^ was evaluated from the Cu LMM Auger spectra. More details on the Cu compound composition analysis are provided in Figure S12. Among the Cu compounds, the percentage of Cu(OH)_2_ was estimated from the O 1*s* intensity corresponding to the Cu–OH bonding. The analysis of the proportion of Cu compounds at the ground and voltage-driven electrodes is summarized in Figure 7a,b, respectively. The most significant change in the proportion of Cu compounds at the ground electrode is the disappearance of Cu_2_O when the external voltage is varied from −1.5 to −3.0 V (from 3A to 4A). Instead, Cu(OH)_2_ appeared (2A to 4A). As shown in the *I*-*V* curve in Figure 4d, humidity strongly affects the electrochemical reaction. Therefore, the water molecule in air is reasonably considered to contribute to the formation of Cu(OH)_2_. The opposite reaction was observed at the counter electrode (voltage-driven electrode), where Cu(OH)_2_ disappeared and Cu_2_O appeared. In terms of CuS_*x*_, the opposite change in proportion, that is, CuS*_x_* decrease at the grounded electrode (3A to 4A) and CuS*_x_* increase at the voltage-driven electrode (3B to 4B), was observed.

The constituent materials on electrode 1A, which was a grounded electrode when the voltage of +3.0 V was applied to counter electrode 1B, were quite analogous to those on electrode 4B. In addition, the constituent materials on electrode 1B were also analogous to those on electrode 4A. Therefore, the electrochemical reactions involving Cu, Cu_2_O, Cu(OH)_2_, CuS_*x*_, and H_2_O may be one of the important reactions causing the Faradaic current and characteristic peak shapes of the *I*-*V* curve. In other words, they can be regarded as information transmitters in the present IL-PRD.

Interestingly, the present XPS experiments show that the Cu oxide is in the Cu_2_O state rather than CuO. The Cu cation valence in Cu(Tf_2_N)_2_ and Cu(OH)_2_ is divalent, but Cu_2_O, a monovalent Cu cation oxide, is particularly formed at negatively biased reaction sites (e.g., 4B and 1A), indicating oxidation/reduction reactions between Cu^+^ and Cu^2+^. One possible pathway for the formation of Cu_2_O is the decomposition of CuF_2_. As mentioned above, in the case of Li(Tf_2_N)/Py1,3(Tf_2_N), Li fluoride was detected.^33^ On the other hand, in the present XPS measurements, no F was detected. This discrepancy may be attributed to the instability of CuF_2_ and fluorocarbon (CF_*x*_) on metallic Cu. Previous studies on the dry etching process of Cu using CF_4_ gas have shown that CuF_2_ and CF_*x*_ on Cu are highly unstable and decompose to Cu_2_O in a high humidity (80%) atmosphere.^34^ The electrochemical reactions at the Cu-IL/Pt interface in the present IL-PRD are strongly influenced by H_2_O, as mentioned earlier. Even if CuF_2_ and CF_*x*_ are formed by the reaction of Cu with CF_*x*_, which are likely the decomposition products of [Tf_2_N]^−^,^28^ they may decompose at the interface in the presence of H_2_O and subsequently form Cu_2_O. The disproportionation reaction between Cu and CuO also forms Cu_2_O in the solid phase.^35^ Such reactions have also been reported for Cu-IL in contact with metallic Cu, where Cu^+^ is formed from Cu and Cu^2+^.^36,37^ As shown in Figure 7, metallic Cu was also detected at the reaction site, which is consistent with the preferential formation of Cu_2_O due to Cu^+^ stabilization. CuS_*x*_ identified by the present XPS is thought to play a significant role in the electrochemical reaction at the reaction sites. According to the previous report,^38^ the Gibbs free energy of formation for Cu_2_S is lower than that of CuS, which is favorable from the viewpoint of stabilizing Cu^+^ to form Cu_2_O. It is reported that the Gibbs free energy difference between Cu_2_S and CuS is only 0.15 V in terms of voltage,^38^ which is much smaller than the external voltage applied to the IL-PRDs in the present study. Therefore, the reverse transition from Cu_2_S to CuS is expected to occur easily during electrochemical measurement. Consequently, the coexistence of Cu, Cu_2_O, and CuS_*x*_ possibly enhances the reproducibility of the electrochemical reactions at the reaction sites because of the reversible redox processes of Cu cations through CuS_*x*_, which reasonably brings about good endurance characteristics, as demonstrated in Figure S6.

Comparing “4A and 5A” in Figure 7a and “4B and 5B” in Figure 7b, it can be seen that the proportion of Cu compounds fluctuates slightly with repeated voltage sweep cycles. This fluctuation in the proportion of Cu compounds leads to a statistical dispersion in the electrical properties of the IL-PRD during repeated operation, which is highly relevant to information processing capabilities. Therefore, the development and optimization of ILs are essential to improve the cycle-to-cycle reparability of chemical reactions at the IL/electrode interface.

### 3.4 Calculation Performances for STM and NARMA2 Tasks

To evaluate the short-term memory (STM) and nonlinear data transformation capabilities of IL-PRD, we discussed the role of Faradaic currents in the physical reservoir computation performance of STM and parity check tasks in the IL-PRD,^15,39^ respectively. Here, we evaluated the performance of IL-PRD by the STM and NARMA2 tasks, well-known benchmarks for PRD, especially for processing time-series data.^21,22^

**Figure 8:**
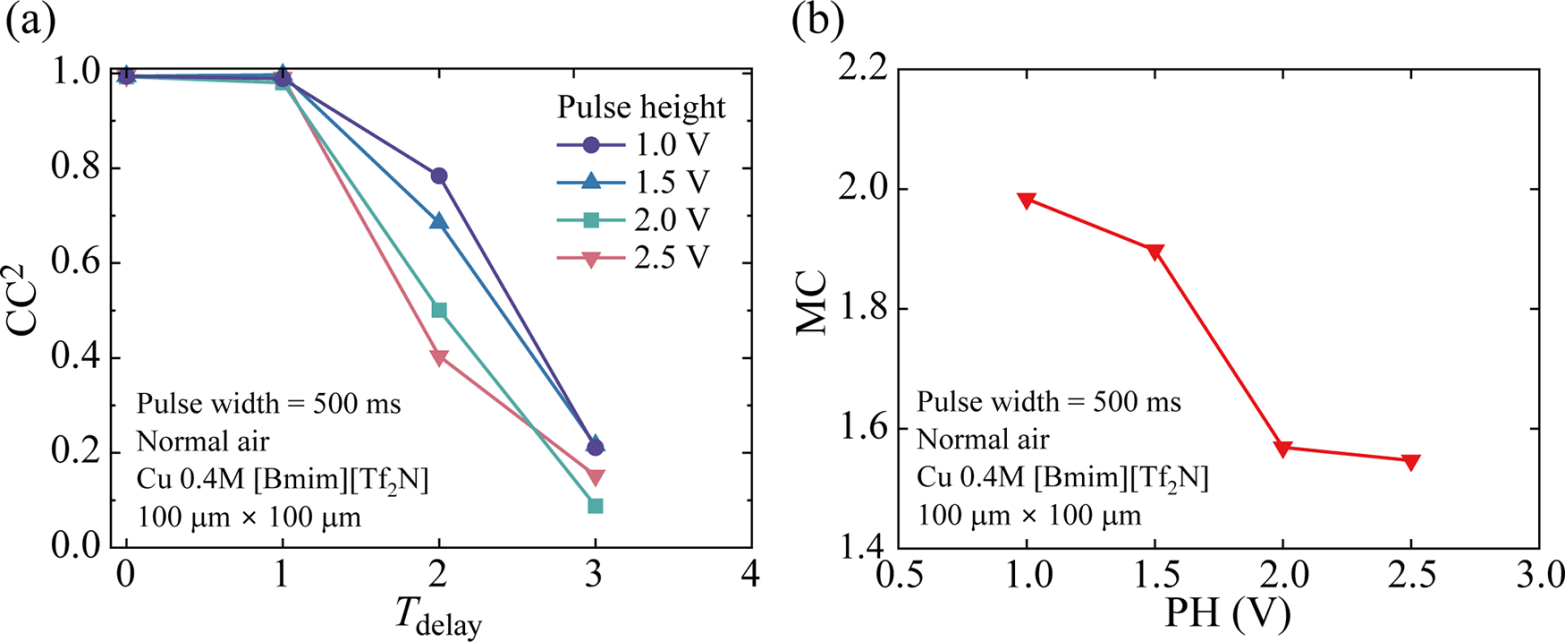
STM task performance for IL-PRD. (a) Squared values of the correlation coefficient (CC^2^) as a function of *T*_delay_, and (b) PH dependence of MC.

In Figure 8, we evaluated the PH dependence of the calculation performance in IL-PRD for the STM task. Here, the value of PH was varied from 1.0 to 2.5 V in analogy with the *I*-*V* curve measurements in Figure 4. The value of PW was fixed to be 500 ms. Figure 8a shows the squared values of the correlation coefficient (CC^2^) for the STM task under various *T*_delay_ conditions. With increasing *T*_delay_, the value of CC^2^ decreased independent of PH. The memory capacity (MC) as a function of PH is plotted in Figure 8b. The value of MC decreased with increasing PH, which is presumably caused by the increase of the cycle-to-cycle variation of the current value with increasing PH. The supplementary explanation about the PH dependence of the MC values in Figure 8b is provided in Figures S13 and S14.

According to the previous research,^40^ the MC value of the STM task is affected by the relationship between the relaxation time of the physical system and the pulse width of the input signal. More specifically, when the pulse width is in a certain appropriate range close to the relaxation time of the physical system, the MC can remain at a relatively large value. In this literature, the authors have clarified that not only too long pulse width but also excessively short pulse width compared to the relaxation time resulted in the decrease in MC and that the appropriate choice of the pulse width for the input signal is essential.^40^ In the present IL-PRD, different from a physical system having a relaxation time scale of 1 μs,^40^ MC remains within the range of 1.5 to 2.0 even when the pulse width is 500 ms, as shown in Figure 8. Such adaptability of IL-PRD to the slowly changing signals is a clear advantage to using electrochemical reactions as the physical dynamics in PRD.

**Figure 9:**
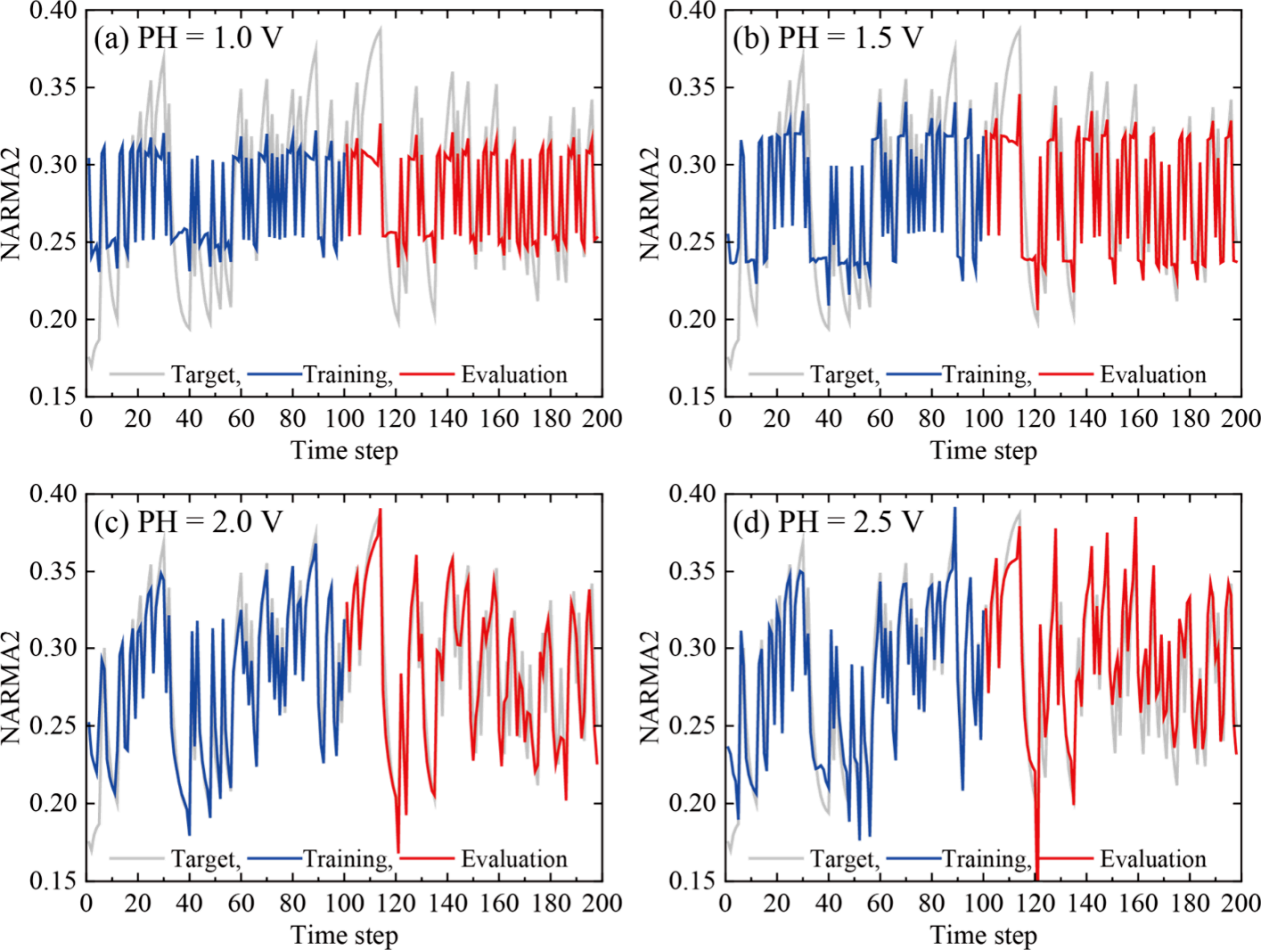
NARMA2 task performance for IL-PRD operated under different voltage pulse conditions: (a) 1.0 V, (b) 1.5 V, (c) 2.0 V, and (d) 2.5 V. The gray, blue, and red curves represent the target signal, the model output signal during the training phase, and the model output signal during the evaluation phase, respectively. Of the 198 data sets, the first 100 data sets were used to train the model, and the last 98 were used to evaluate the training results.

Figure 9 shows the performance of the NARMA2 task during the training (blue) and evaluation (red) phases, evaluated by using the output signals of IL-PRDs operating at different PHs. The target signal for each time step is also depicted (gray). As shown in Figure 9a, at PH = 1.0 V, the changing trend of the target signal is qualitatively reproduced. However, the amplitudes between the target and the evaluation signals are different. As PH increases, the target signal is more accurately traced by the evaluation signal, as shown in Figure 9d. The evaluation results using the 298 data sets are shown in Figure S15. Also, we evaluated the influence of the training data number on the NARMA2 task performance in the evaluation phase, which is also provided in Figure S16. In addition, the influence of the virtual node number *k* on the NARMA2 task performance in the evaluation phase was also investigated, which is shown in Figure S17.

**Figure 10:**
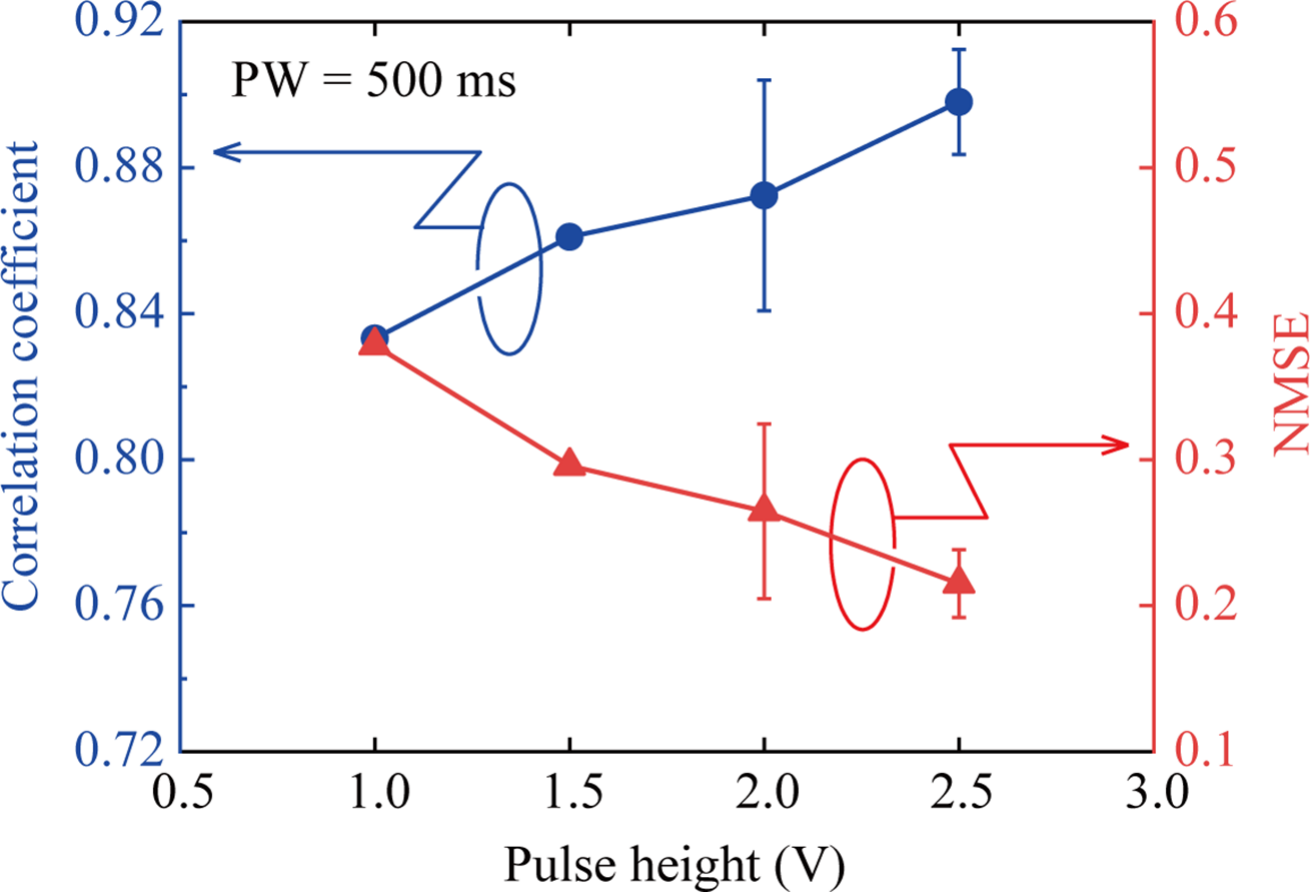
Pulse height dependence of correlation coefficients (left axis) and NMSE values (right axis) evaluated for the NARMA2 task performance.

The performance analysis results (CC and NMSE) for the NARMA2 task are plotted in Figure 10 as a function of PH. In this study, machine learning for the NARMA2 task was repeated five times for each PH condition, and the average values of CC and NMSE are plotted in Figure 10. The error bars in Figure 10 are displayed based on the maximum and minimum values. As PH increases, the value of CC increases and the value of NMSE decreases monotonically. As shown in Figure 7, the proportion of Cu compounds changes more drastically with increasing PH. At the same time, the subsequent Faradaic current intensity and hysteresis of the *I*-*V* curve increase with increasing PH, as shown in Figure 4. Thus, the output signal from the IL-PRD has a more pronounced time correlation and nonlinear transformation capability with respect to the input signal, leading to improved performance in the NARMA2 task.

Different from the PH dependence of the MC value in the STM task shown in Figure 8b, the value of CC in the NARMA2 task increased with PH, which indicates that the hysteretic and nonlinear transformations of the input TVPs by IL-PRD improved the NARMA2 task performance. Actually, as shown in Figure S18, the output signal from a resistor, which generated the linearly transformed output signal, significantly decreased the NARMA2 performance. We also conducted the NARMA2 task using a software-based long–short-term memory (LSTM) network, which is one of the conventional RNNs. The output current from a resistor was used for the input signal to the LSTM network. By using the LSTM network, the CC value in the NARMA2 task comparable to that obtained by using IL-PRD was obtained (Figure S19). However, in order to implement the LSTM unit by physical devices, a large number of the resistance switching devices (memristors) are required.^41,42^ Therefore, the introduction of IL-PRD having hysteretic and nonlinear transformation characteristics leads to the size reduction of the PRC system, which is favorable for the application in the field of edge computing.

The electrochemical properties of IL-PRD can be controlled by the device structure such as the electrode area and the material properties of the ionic liquid such as the metal ion concentration, considering that the waveform of the cyclic voltammogram depends on the ohmic drop and the concentration of the redox species.^43^ Therefore, through optimization of these parameters regarding the device structure and ionic liquid, the information processing capability of IL-PRD is expected to improve further. In addition, as reported in some other physical reservoir devices,^44,45^ the increase of the PRD number is considered to be one of the promising ways to improve the information processing capability of the PRC system using IL-PRD. The plausible IL-PRD structure with multiple electrodes is provided in Figure S20.

In the present study, the thickness of the IL droplet is not controlled exactly, which is thought to cause variation in the solution resistance between the input and output electrodes because the cross-sectional area of the droplet depends on the thickness of the IL droplet. However, as shown in Figures S21 and S22, in the present IL-PRD, the influence of the variation in the solution resistance on the electrical property was negligibly small. Therefore, the present IL-PRD is reasonably expected to have robustness against variation in the IL droplet thickness and consequent variation in the solution resistance. To ensure the accuracy in the thickness of the IL droplet, the introduction of the liquid encapsulation technology on a microfabricated circuit is thought to be effective, which has been demonstrated in the literature on the liquid memory technology.^46^

### 3.5 Demonstration of ECG Signal Classification Task

**Figure 11:**
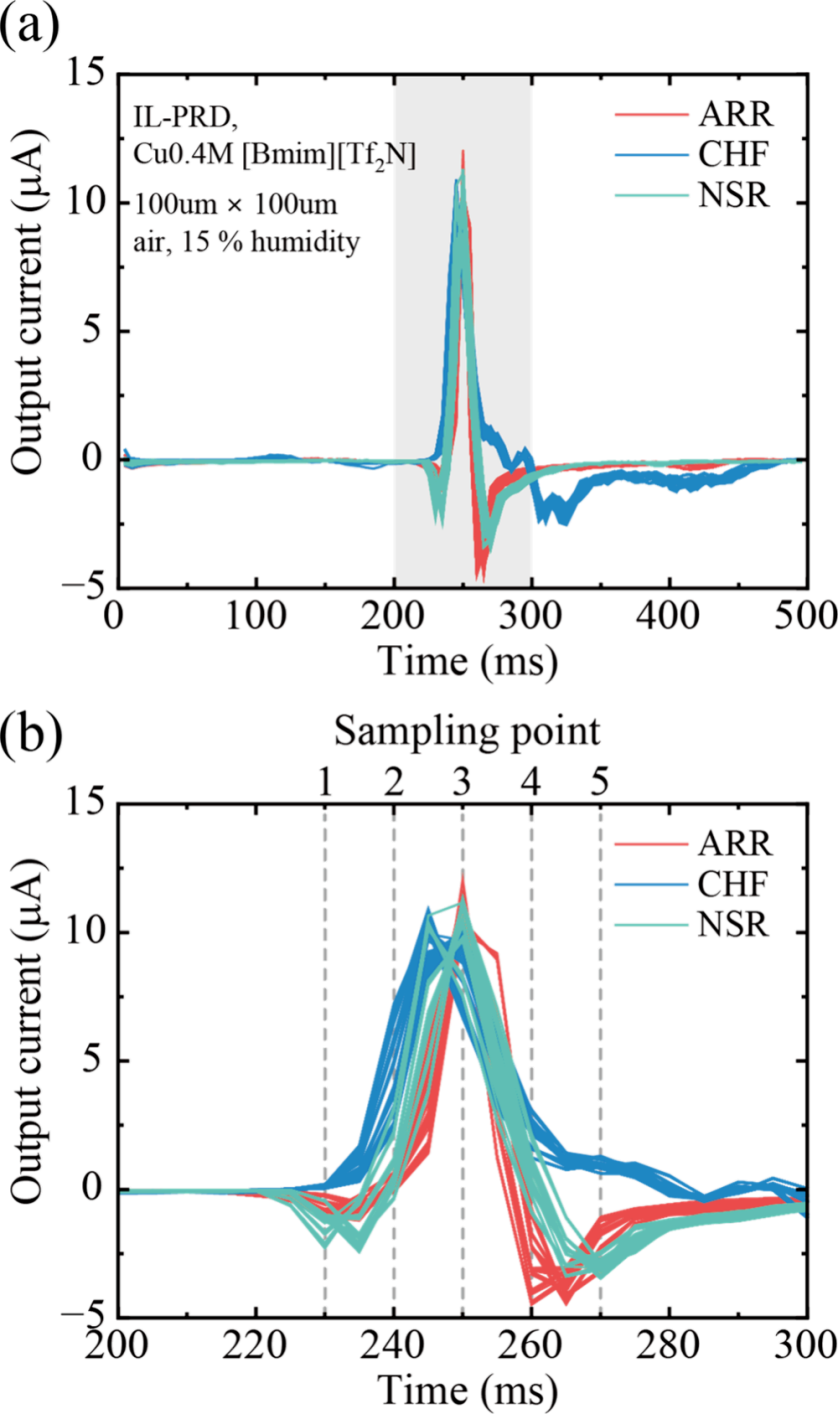
(a) Output current waveforms from IL-PRD when ARR, CHF, and NSR signals were input as a volage pulse. (b) Expansion of the gray shaded region in (a) and sampling points to acquire the output current data set to feed to the virtual node for machine learning. The humidity of the air was approximately 15% when these output currents were measured.

**Figure 12:**
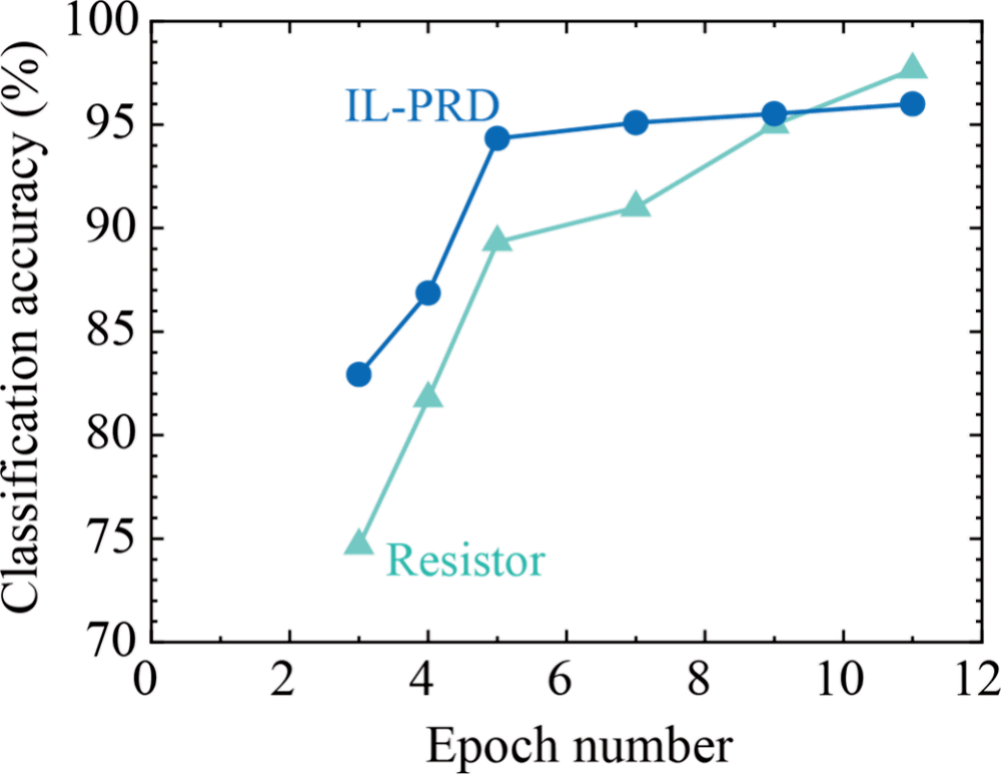
Epoch number dependence of the classification accuracy for three classes of the ECG signals when the output current data set from five sampling points were used for the machine learning.

The importance of PRC under a limited data number was pointed out from the viewpoint of the energy efficiency in the RC hardware.^47^ Therefore, in the present study, we evaluated the ECG signal classification accuracy in IL-PRD when the number of virtual nodes was limited. Figure 11a shows the output current waveform from IL-PRD measured when the ECG signals (Figure S5) were applied. Figure 11b shows the output current before and after the peak at around 250 ms, which corresponds to the gray-shaded region in Figure 11a. The sampling points to obtain the output current data set to feed to the 5 virtual nodes are also depicted in Figure 11b. In the present study, we evaluated the dependence of the classification accuracy on the epoch number for the training process of the neural network because the increase in the epoch number causes additional energy consumption for the information processing. The calculation process of the classification accuracy is explained in Figure S23. The epoch number dependence of the classification accuracy is summarized in Figure 12. For comparison, the classification accuracy when the output current from a resistor, which has a waveform identical to the input ECG signal, is also plotted. Compared with the case of the resistor, the output current data set from IL-PRD clearly increased the classification accuracy even under the condition of the small epoch number, which is expected to reduce the information processing energy.

**Figure 13:**
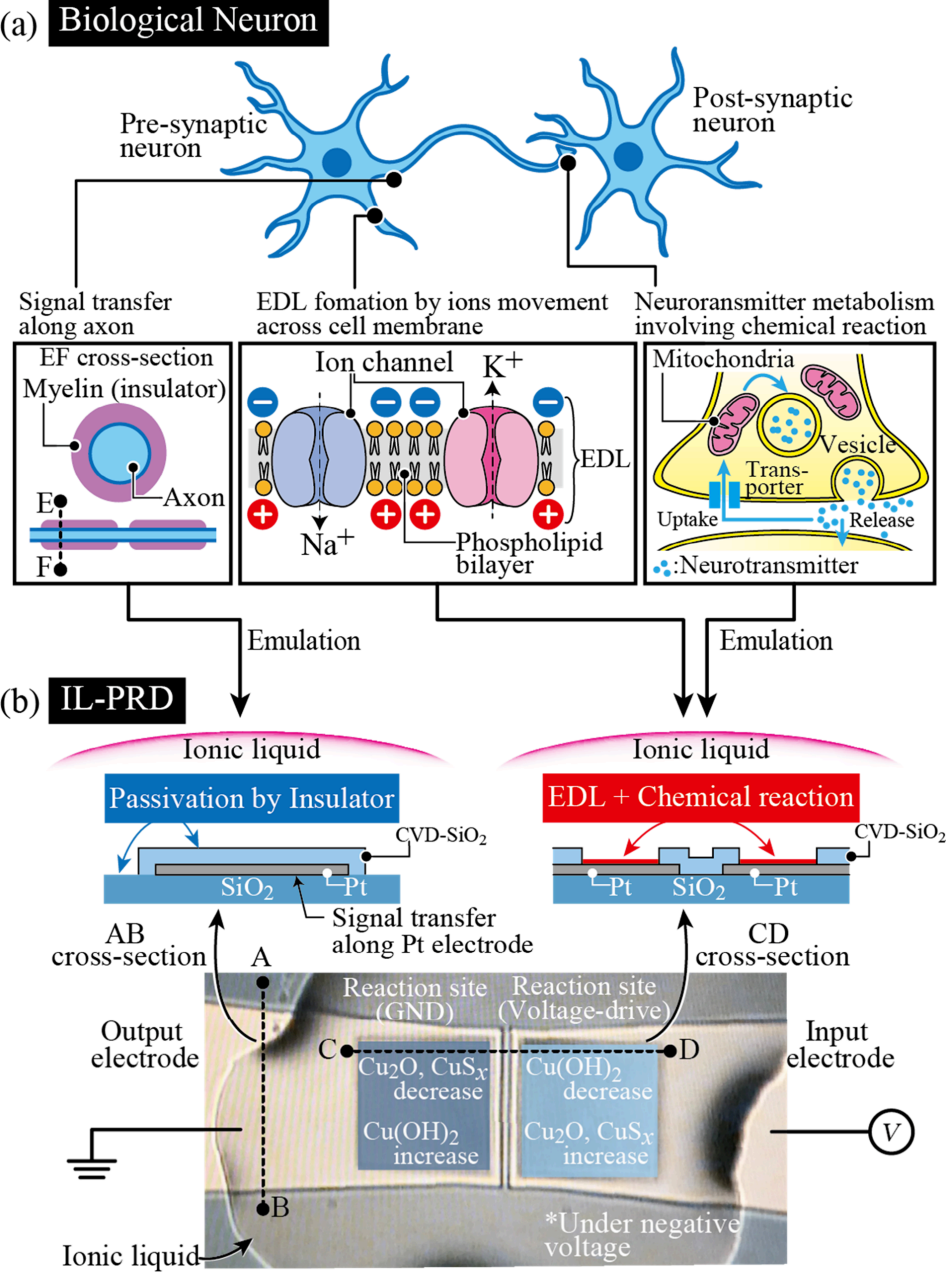
Analogy between (a) biological neurons and (b) IL-PRD. (a) Schematic showing axons surrounded by myelin (an insulator), an electrical double layer (EDL) formed on a phospholipid bilayer, and neurotransmitter metabolism. These three are fundamentally associated with the signal transfer between pre- and postsynaptic neurons. (b) Schematic showing that IL-PRD multilaterally emulates the mechanism of signal transfer in biological neurons. Similar to neurotransmitter metabolism, reproducible electrochemical reactions involving Cu, Cu(OH)_2_, Cu_2_O, CuS_*x*_, and water molecules at the reaction sites generate Faradaic currents, which strongly influence the capability of IL-PRD to process time-series data.

Figure 13 shows the similarities between biological neurons (Figure 13a) and the present IL-PRD (Figure 13b). There are some similarities between the physical structures of biological neurons and IL-PRDs. For instance, as the axons are surrounded by myelin (an insulator) to transfer action potentials farther,^48^ the input and output electrodes of the IL-PRD are surrounded by CVD-SiO_2_ to avoid unfavorable Cu deposition outside the reaction sites. Also, as a synaptic gap separates presynaptic and postsynaptic cells,^49^ IL separates the two reaction sites in IL-PRD. Thus, biological neurons and IL-PRD share the elementary processes of electrical signal transmission: charging and discharging EDLs and chemical reactions. In the biological neuron shown in Figure 13a, EDLs are formed and annihilated at the cell membrane surface by the movement of cations (Na^+^ and K^+^) and anions (Cl^–^) in intracellular and extracellular fluids, and voltage pulses are propagated to the synapses. As a result, neurotransmitters are emitted from synaptic vesicles in the presynaptic neurons.^49^ The hydration and dehydration processes of water molecules are closely related to the selectivity of ion channels and thus play an important role in the movement of Na^+^ and K^+^.^50^ The emitted neurotransmitter activates receptors in postsynaptic neurons, causing the chemical signals of the neurotransmitters to be retranslated into voltage impulses in the postsynaptic neurons. Besides, the circulation of neurotransmitters is influenced by water. For instance, acetylcholine (ACh), a well-known neurotransmitter, is hydrolyzed to acetic acid and choline in the presence of the ACh-degrading enzymes.^51^ Finally, the decomposed products are taken up by presynaptic neurons, where ACh is resynthesized. In other words, water plays a role as important as that of metal ions and neurotransmitters. In the case of the IL-PRD shown in Figure 13b, the input voltage signal induces the rearrangement of ions in the IL at the IL/metal electrode interface, and the large electric field in the ELD triggers electrochemical reactions involving water. This can be regarded as a basic functional emulation of biological neurons in terms of information transmission.

Because of its higher-order similarity to biological neurons, we believe that IL-PRD is very suitable for processing time-series signals associated with vital activities of humans, which is successfully demonstrated using the ECG signal classification task in Figure 12. The efficiency of RC for the recognition of human state and activity has previously been pointed out by a software simulation.^52^ The time scales of the signals derived from the human state and activities are quite long compared to the operating speed of some advanced electronic devices,^44,45^ while it is quite analogous to the operating speed of IL-PRD. Therefore, not only for the ECG signal classification task but also for the recognition tasks of various signals relevant to the human state and activity, IL-PRD is expected to extract the features of those signals efficiently, which accelerates the implementation of low-power AI processors suitable for edge AI processing.

## 4 Conclusions

We successfully developed IL-PRD by using electrochemical reactions in ILs containing Cu cations. Due to Faradaic currents, highly diverse output signals are successfully generated from IL-PRD, which is adequate as feature-extracted signals for processing in machine learning algorithms such as linear regression. The origin of the electrochemical reactions that produce Faradaic currents in IL-PRD was investigated by XPS, and Cu, Cu_2_O, Cu(OH)_2_, CuS_*x*_, and H_2_O were identified as the dominant reactants that can be regarded as information-transmitting materials in the device. Since the proportion of information-transmitting materials on the electrodes changes slightly with repeated device operation, it is essential to realize the reproducibility of electrochemical reactions in terms of reliable operation of IL-PRD. Time-series data processing capability of the STM and NARMA2 tasks was evaluated. An information processing capability for the NARMA2 task was improved with nonlinear Faradaic currents. Moreover, the efficiency of IL-PRD for the ECG signal classification task was demonstrated. To further improve the performance of IL-PRDs, it is essential to synchronize the development of IL materials, PRD structures, and device fabrication processes. To realize the practical device implementation of RC using IL-PRD, it is required to increase the compatibility with the CMOS processes much further. When this progress is achieved, IL-PRD will ultimately promote AI implementation at the real-world edge computing domain.

## Supporting Information Available

Photographs of the prober system used in the present experiment, photograph of the gas purge system to prepare the sample for the XPS measurement, estimated net detection area in the present XPS measurement, summary of the STM and NARMA2 task evaluation processes for IL-PRD, ECG signal waveforms used in the classification task, cycle endurance test results up to 5000 cycles of the voltage pulse application, C 1*s*, N 1*s*, O 1*s*, and S 2*p* XPS spectra at the reaction sites in the grounded and voltage-driven electrodes, Cu 2*p* 3/2 XPS spectrum measured using the control sample, examples of the waveform separation analysis for the Cu 2*p* XPS and Cu LMM Auger spectra, percentage of areal intensities of component waves in Cu 2*p* XPS and Cu LMM Auger spectra together with the amount of Cu, O, and S, estimated ideal STM performance as a function of the voltage pulse height, NARMA2 task performance evaluated using 100 data sets for training and 298 data sets for evaluation, influence of the training data number on the correlation coefficient for the evaluation phase of the NARMA2 task, virtual node number dependence of the NARMA2 task performance, NARMA2 task performance using output current data set from a resistor and linear regression, NARMA2 task performance using output current data set from a resistor and long–short-term memory (LSTM) network, photograph of the IL-PRD with multiple electrode structure, and effects of the solution resistance on the electrical property of IL-PRD (PDF)
