# Supplementary material for: Characterization of Information-Transmitting Materials Produced in Ionic Liquid-based Neuromorphic Electrochemical Devices for Physical Reservoir Computing
Source: ACS Appl Mater Interfaces. 2023 Oct 10;15(42):49712–26. doi: 10.1021/acsami.3c08638 (PMC10614198; doi:10.1021/acsami.3c08638)
Supplement: Supplementary file 1 — am3c08638_si_001.pdf [file am3c08638_si_001.pdf]

Supporting Information
Characterization of Information-Transmitting Materials Produced in Ionic Liquid-based Neuromorphic Electrochemical Devices for Physical Reservoir Computing

Dan Sato[1, 2], *Hisashi Shima*[1,*], *Takuma Matsuo*[1, 2], *Masaharu Yonezawa*[1, 2], *Kentaro Kinoshita*[2, *], *Masakazu Kobayashi*[2, 3], *Yasuhisa Naitoh*[1], *Hiroyuki Akinaga*[1], *Shunsuke Miyamoto*[4], *Toshiki Nokami*[4], and *Toshiyuki Itoh*[5]

[1] Device Technology Research Institute, National Institute of Advanced Industrial Science and Technology, Tsukuba, Ibaraki 305-8565, Japan

[2] Department of Applied Physics, Graduate School of Science, Tokyo University of Science, Katsushika, Tokyo 125-8585, Japan

[3] New Value Creation Office, NAGASE & CO., LTD., Nihonbashi, Tokyo 103-8355, Japan

[4] Center for Research on Green Sustainable Chemistry, Faculty of Engineering, Tottori University, Koyama, Tottori 680-8552, Japan

[5] Toyota Physical and Chemical Research Institute, Nagakute, Aichi 480-1192, Japan

*Corresponding authors: Hisashi Shima (e-mail: shima-hisashi@aist.go.jp) and
Kentaro Kinoshita (e-mail: kkinosita@rs.tus.ac.jp)

Table of Contents

Figure S1	Photographs of the prober system.	SI4
Figure S2	Photographs of desiccator with gas replacement function.	SI4
Figure S3	Optical microscope image of IL-PRD for XPS measurement.	SI 5
Figure S4	Summary of the STM and NARMA2 task evaluation processes for IL-PRD...	SI 6
Figure S5	ECG signal waveforms used in the classification task	SI 7
Figure S6	Cycle endurance test results for IL-PRD.	SI 8
Figure S7	C 1*s* XPS spectra of the reaction sites.	SI 9
Figure S8	N 1*s* XPS spectra of the reaction sites.	SI10
Figure S9	O 1*s* XPS spectra of the reaction sites.	SI10
Figure S10	S 2*p* XPS spectra of the reaction sites.	SI11
Figure S11	Cu 2p 3/2XPS spectrum of the control sample.	SI11
Figure S12	Examples of the waveform separation analysis for the Cu 2*p* XPS and Cu LMM Auger spectra	SI12
Table S1	Percentage of areal intensities of component waves in Cu 2*p* XPS and Cu LMM Auger spectra together with the amount of Cu, O, and S	SI13
Figure S13	Color-coded current-voltage curves for raw and averaged data.	SI14
Figure S14	Memory capacity (MC) for STM task evaluated using averaged data.	SI15
Figure S15	NARMA2 task performance with 100 training and 298 evaluation datasets....	SI15
Figure S16	Training data number dependence of NARMA2 task performance.	SI16

Figure S17 Virtual node number dependence of NARMA2 task performance................... SI16

Figure S18 NARMA2 task performance using output current signal from a resistor.......... SI17

Figure S19 NARMA2 task performance using long-short-term memory network............. SI17

Figure S20 An example of the confusion matrix for the ECG signal classification task…. SI18

Figure S21 Optical microscope image of IL-PRD with multiple electrodes....................... SI19

Figure S22 Optical microscope image of IL-PRD to evaluate the effect of solution resistance by changing the spacing between input and output electrodes......... SI19

Figure S22 Comparison of the current-voltage curves when the the spacing between input and output electrodes was changed……………………………………….. SI20

Figures S1(a) and S1(b) are the photographs of the outer and inner appearance of the prober system used in the present experiment, which has the gas-exhausting and gas-introduction functions. As shown in Figure S1(c), the W probes can contact on the contact pads of the device under test. Also, since this prober system has vacuum pumps (turbo-molecular pump and scroll pump), the atmosphere during the electrical measurement is variable from uncontrolled normal air to the precisely controlled atmosphere such as high-vacuum and synthesized dry-air.

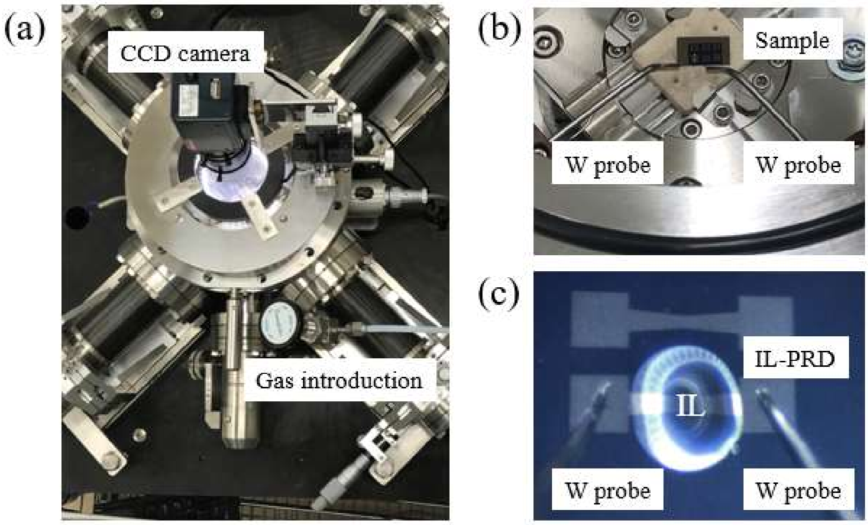

Figure S1. Photographs for (a) outer appearance and (b) inner appearance of the prober system. (c) Screen of the attached monitor showing the device under test.

Figure S2 is a setup to transfer the sample for the XPS measurement after washing out the IL droplet. The desiccator was filled by a high purity nitrogen gas whose pressure was higher than the atmospheric pressure. Before introducing the nitrogen gas into the desiccator, internal gas (normal air) was removed by using a high-vacuum pump.

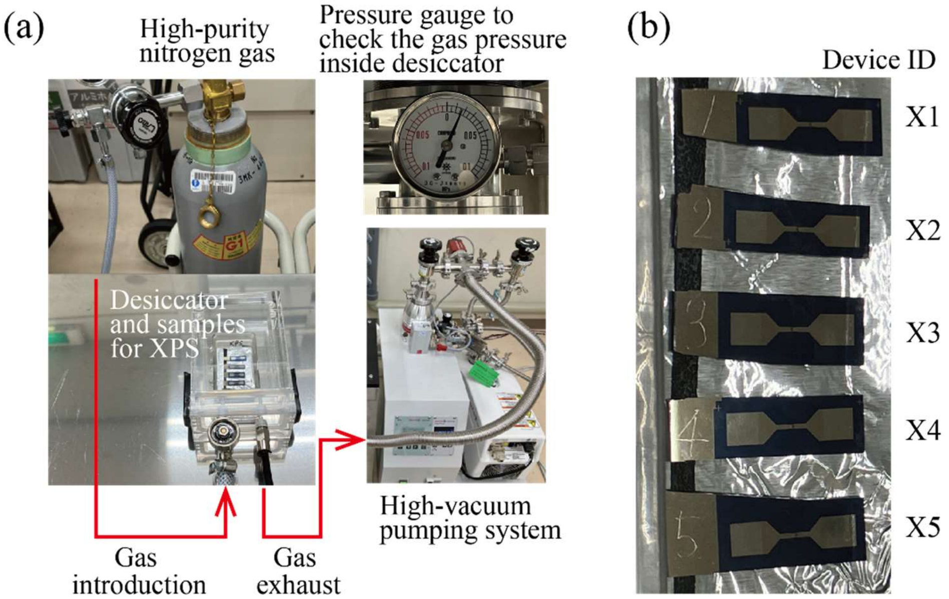

Figure S2. Experimental setup to transfer the device for XPS measurement to the XPS measurement system. (a) The desiccator and the samples for XPS as well as the gas introduction and gas exhaust functions. (b) The optical microscope image of the devices for the XPS measurement taken from outside of the desiccator.

The red circles depicted in Figure S3 are the estimated net detection area in the present XPS measurement. When the right electrode is analyzed, the signal from the left electrode is not detected.

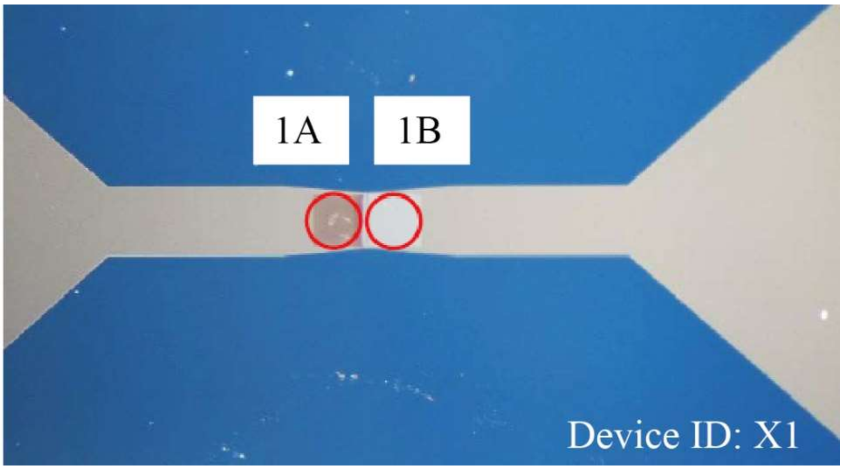

Figure S3. The optical microscope image of IL-RPD for the XPS measurement. Red circles correspond to the net detection area.

Figure S4 is the summary of the STM and NARMA2 task evaluation processes for IL-PRD from input voltage application to linear regression analysis with output current values. In both the STM and NARA2 tasks, the feature vector $I(T)$ of $y(T)$ is generated from the output current values from the IL-PRD by applying the TVPs corresponding to $u(T)$. Specifically, the value of PH for $u(T) = 0$ is the same as that for $u(T) = 1$, whereas the signs of those TVPs are different from each other: negative for $u(T) = 0$ and positive for $u(T) = 1$. At each time step T (i.e., each TVP), a total of 50 data points of output current values $i_k(T)$ ($k = 1, 2, \ldots, 50$) were sampled at the same time interval. Namely, $I(T)$ can be expressed as $I(T) = (i_1(T), i_2(T), i_3(T), \ldots, i_{50}(T))^{Tr}$. In other words, the one-dimensional time-series data of the input voltage $u(T)$ is mapped into a higher dimensional space consisting of the output current $i_k(T)$ by IL-PRD. This so-called "virtual node" technique enables PRC with a single PRD [4]. Then, the output current value $i_k(T)$, obtained as the output signal from the k-th virtual node, was used for linear regression with $y(T)$ as the target value. The least-squares error function was optimized by stochastic gradient decent to train the linear regression model. All linear regression calculations in this study were performed in MATLAB (version R2021a, Mathworks).

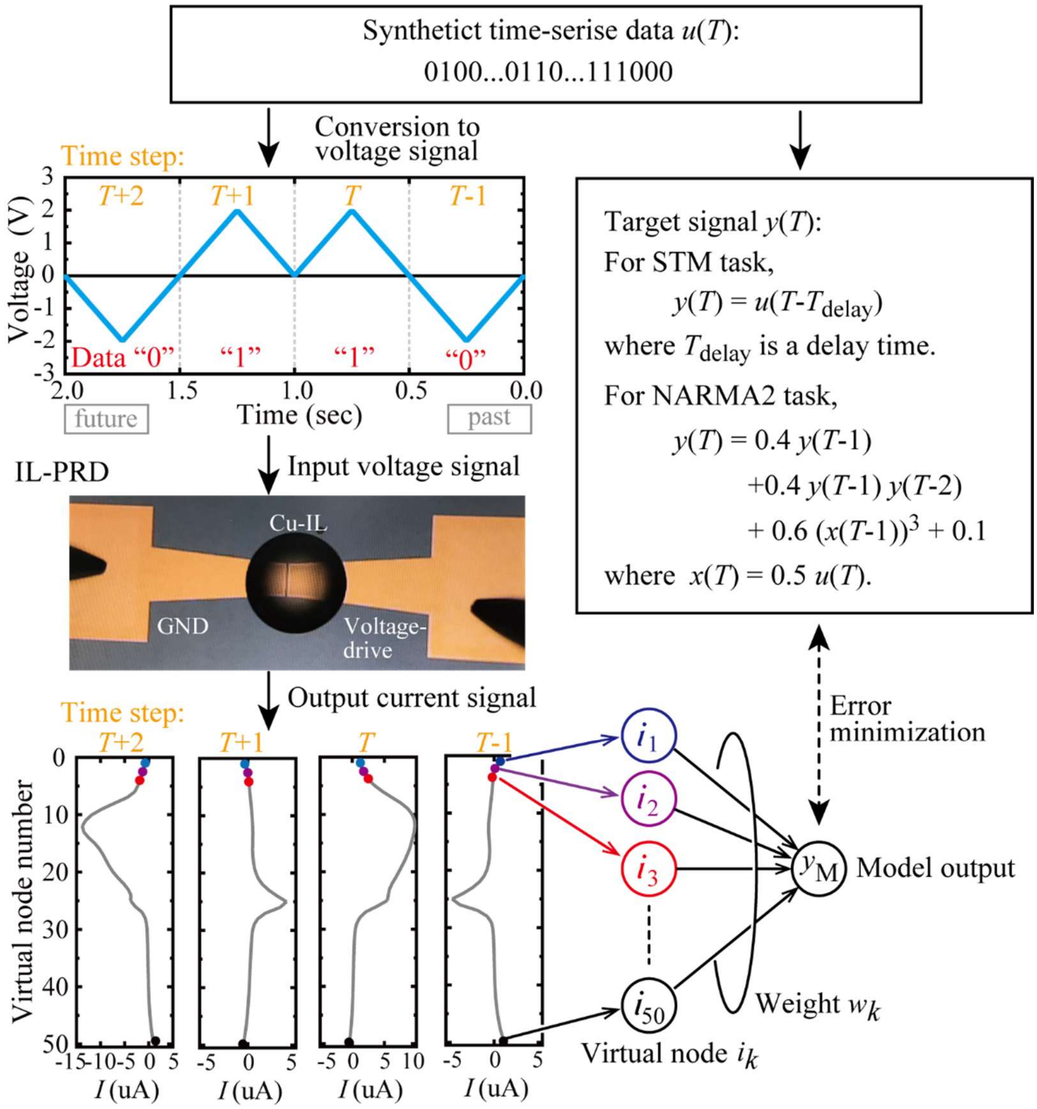

Figure S4. STM and NARMA2 task evaluation processes from applying an input voltage to the IL-PRD to linear regression analysis with output current values. A sequence of randomly selected binary data (0 and 1) was transformed into a triangular voltage pulse train and applied to the IL-PRD. Then, a virtual node method with node number k = 50 was applied to create an input dataset for linear regression.

Figure S5 is the ECG signal waveforms used in the signal classification task. The value of PH was adjusted based on the operating volage of IL-PRD. The value of PW was fixed to be 500 ms.

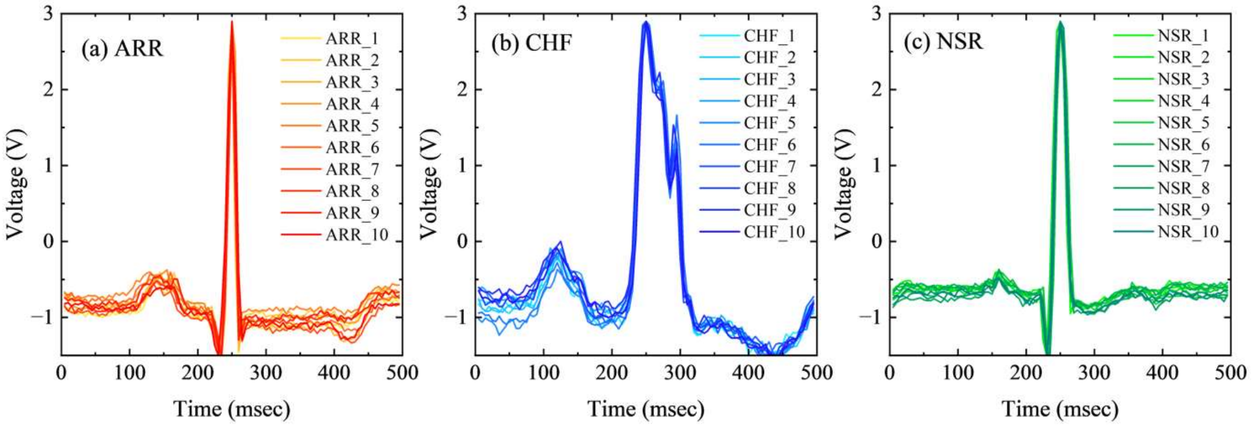

Figure S5. ECG signal waveforms corresponding to (a) ARR (arrythmia), (b) CHF (congestive heart failure), and (c) NSR (normal sinus rhythm) state. The original data to prepare the present ECG signal waveform was downloaded from the repository [23].

Figures S6(a), (c), and (e) are the current-voltage curves up to the 5,000 cycles of the triangular voltage pulse (TVP) application. The value of the pulse width (PW) was varied from 100 ms to 500 ms. The pulse heigh (PH) was fixed to be +2.3 V for the positive TVP and -2.3 V for the negative TVP. Figures S6(b), (d), and (f) are the pulse number dependence of the Faradaic current intensity when PW = 100, 300, and 500 ms, respectively. The read-out voltage (V_{read}) was ±1.0 V. The endurance characteristics is influenced by the condition of the applied TVPs. For PW = 100 ms, the degradation of the Faradaic current was not observed. On the other hands, for PW = 500 ms, the Faradaic current intensity decreased with increasing the number of TVP. Every 200 TVP application, the latency time for the data transfer inside the measurement system is unintentionally introduced. The electrode surface state and/or the Cu ions concentration near the electrode surface seems to change slightly during the latency time because the cycle-to-cycle current continuity breaks every 200 TVPs, although there was no appearance change of the reaction sites. Overall trend of the cycle number dependence of the Faradaic current intensity can be analyzed regardless such discontinuity.

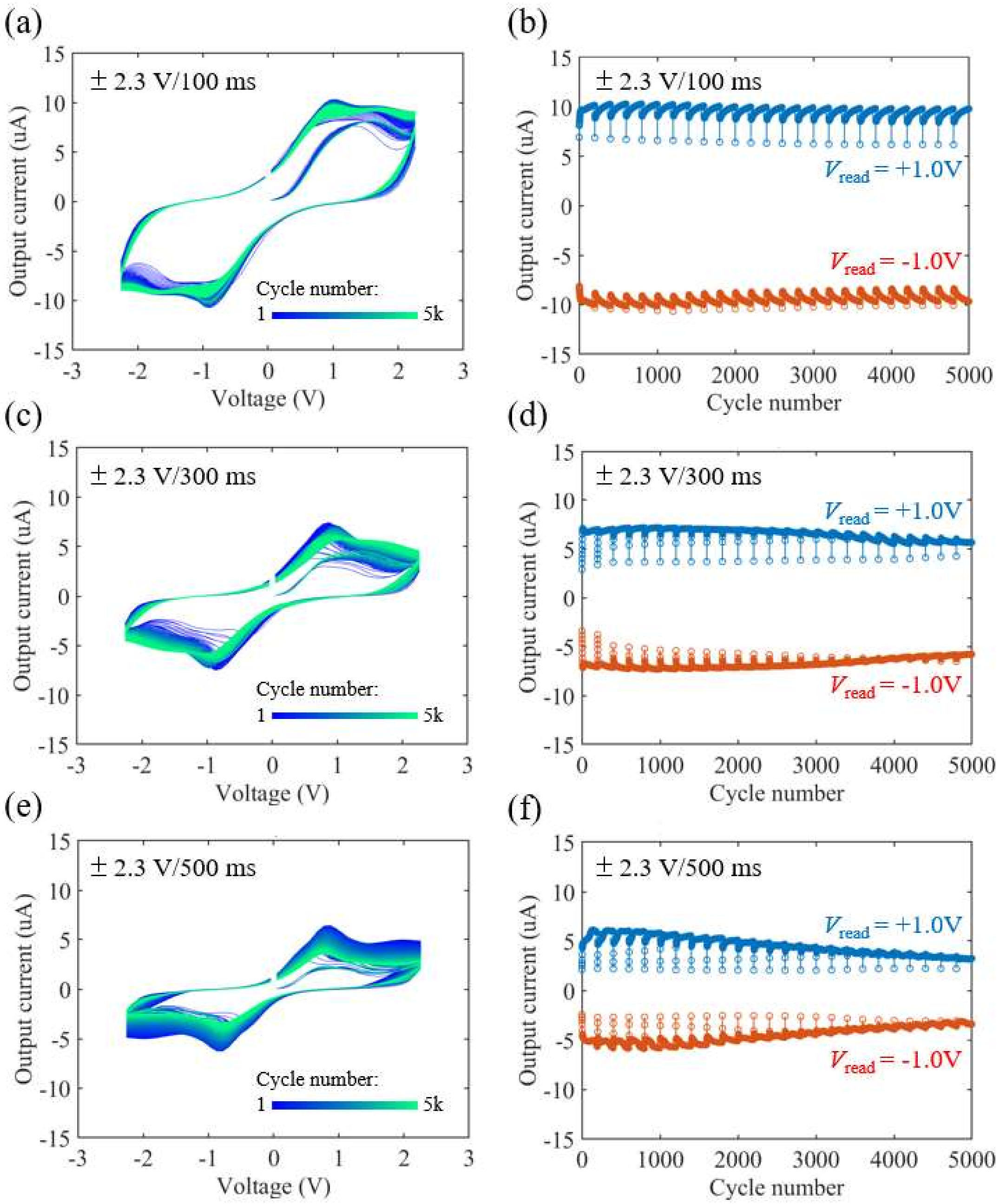

Figure S6. Current-voltage curves up to 5000 cycles of the TVP application when the pulse width (PW) is (a) 100 ms, (c) 300 ms, and (e) 500 ms. The cycle number dependence of the Faradaic current peaks (current values read at ±1.0 V) when PW is (b) 100 ms, (d) 300 ms, and (f) 500 ms. The value of PH was fixed to be ±2.3 V.

Figures S7-S10 are the C 1*s*, N 1*s*, O 1*s*, and S 2*p* XPS spectra of the reaction sites at the ground and voltage-drive electrodes, respectively. In the C 1*s* XPS spectra in Figure S5, no peak related to $CuCO_3$ (i.e., chemical bonding of C=O or O=C-O at binding energies between 289 and 290 eV) was observed. In Figures S6, nitrogen was detected at the GND electrode 1A and at the volage-drive electrodes 3B and 4B, where the voltage of -3 V was applied. Therefore, these signals of nitrogen are thought to be derived from a decomposition of $[Tf_2N]^-$. Very weak signals of Ta 4f detected at the electrodes 4A, 5A, 1B, and 2B may correspond to Ta at the edge of the electrodes (peripheral region) and/or Ta implanted in the Pt layer by Ar ions during the Ar milling process. Ta is thought to be in the oxidized state, and therefore the influence on the electrochemical reactions is negligible. Also, from the O1*s* XPS spectra in Figure S9, a prominent sing of Cu-OH and Cu-O bonding was observed (blue- and yellow-highlighted binding energy ranges). In terms of the chemical bonding state between Cu and S, CuS_x rather than $CuSO_x$ is considered to be predominant according to the S 2*p* XPS spectra in Figure S10.

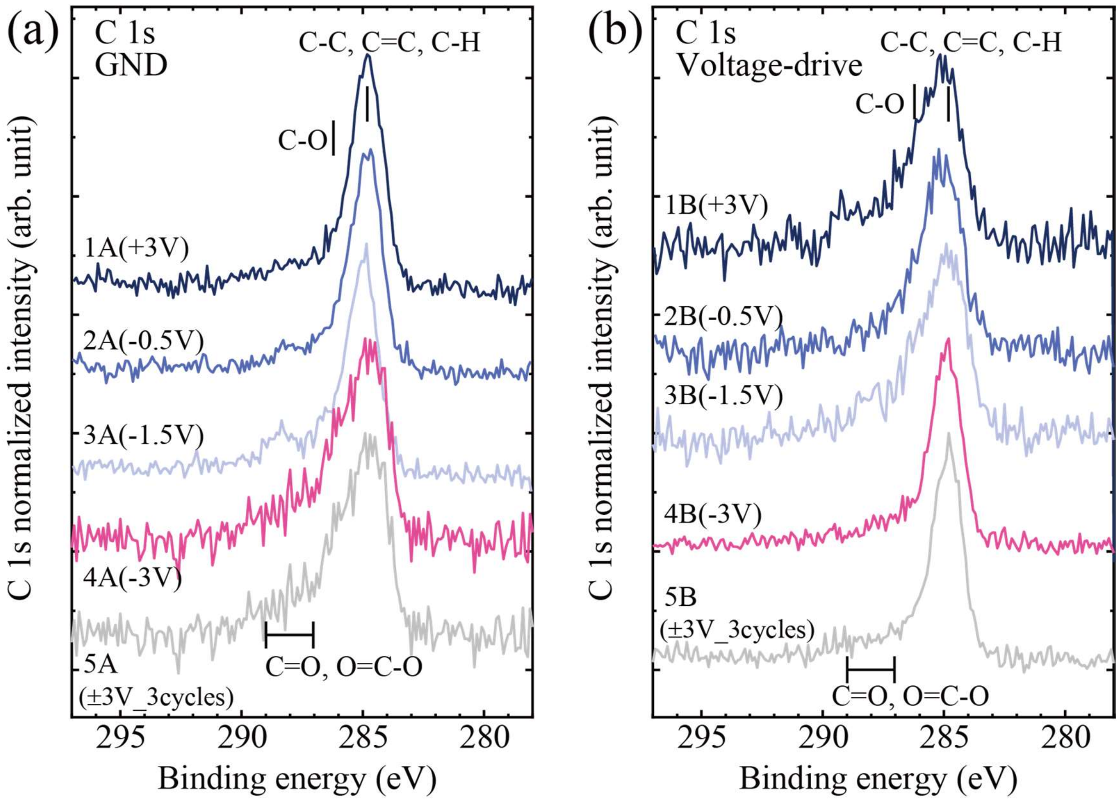

Figure S7. C 1*s* XPS spectra of the reaction sites at the (a) grounded and (b) voltage-drive electrodes.

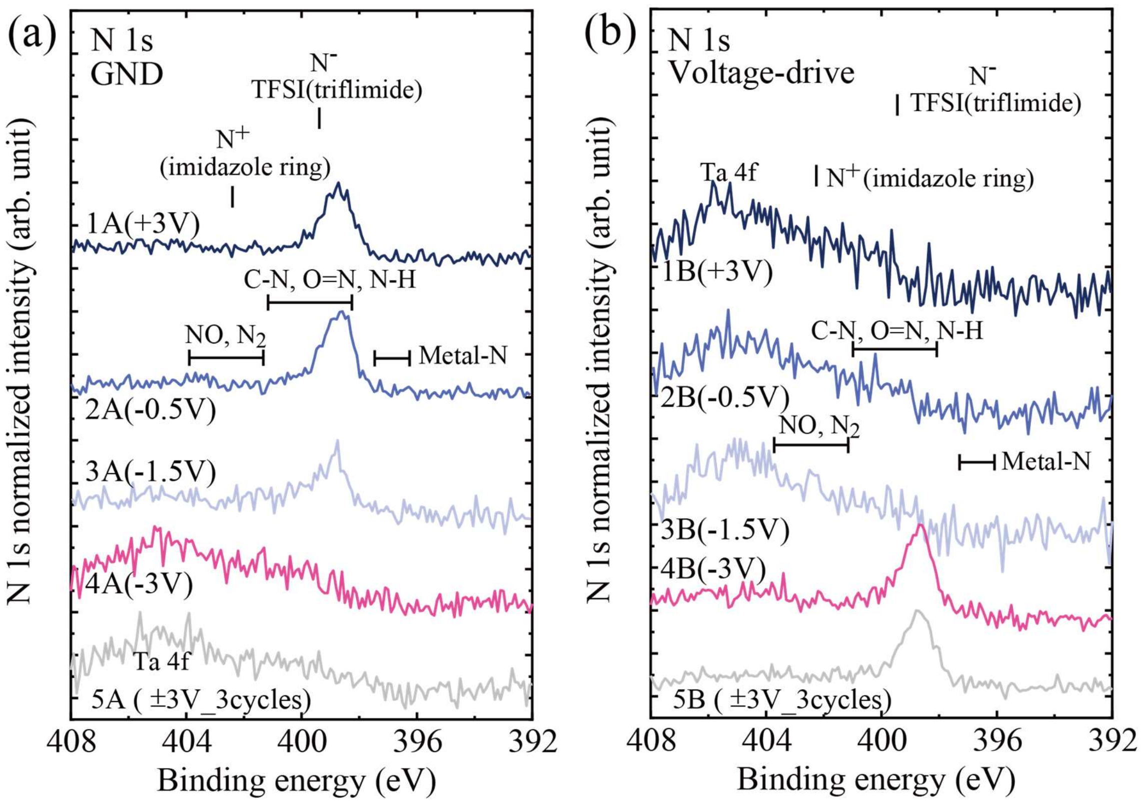

Figure S8. N 1*s* XPS spectra of the reaction sites at the (a) grounded and (b) voltage-drive electrodes.

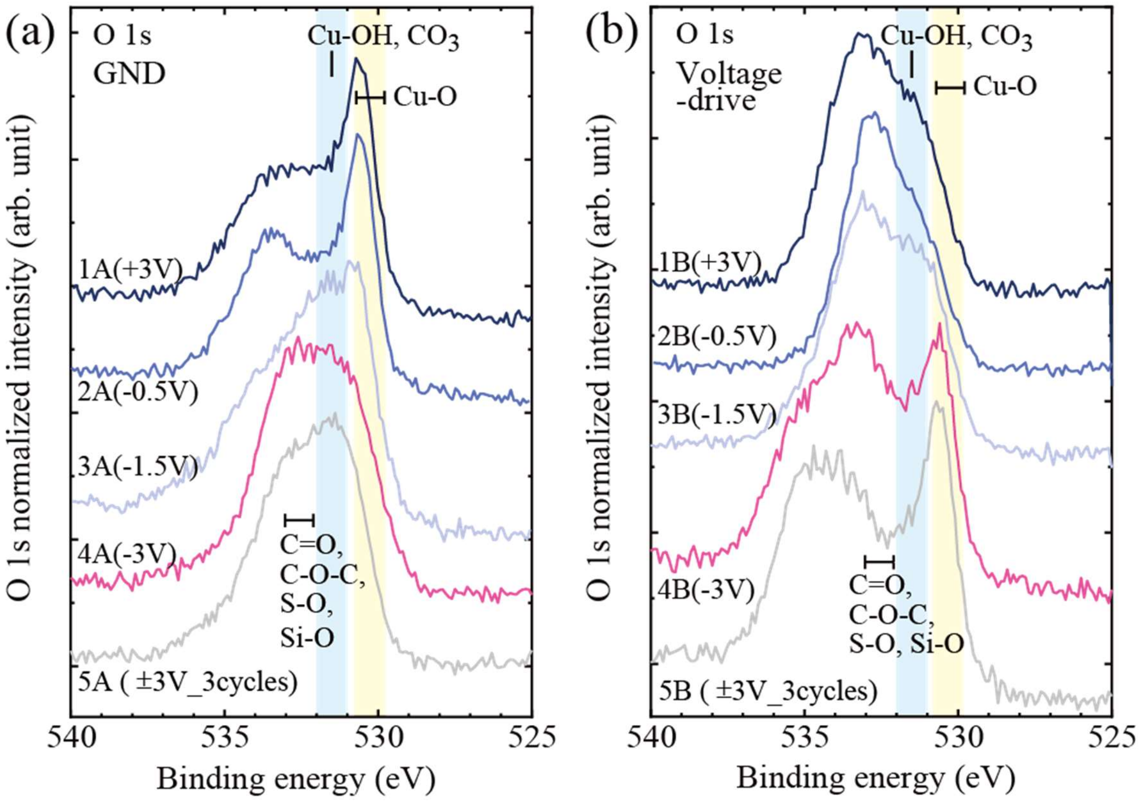

Figure S9. O 1*s* XPS spectra of the reaction sites at the (a) grounded and (b) voltage-drive electrodes. The binging energy ranges corresponding to the Cu-O and Cu-OH bond are yellow-highlighted and blue-highlighted, respectively.

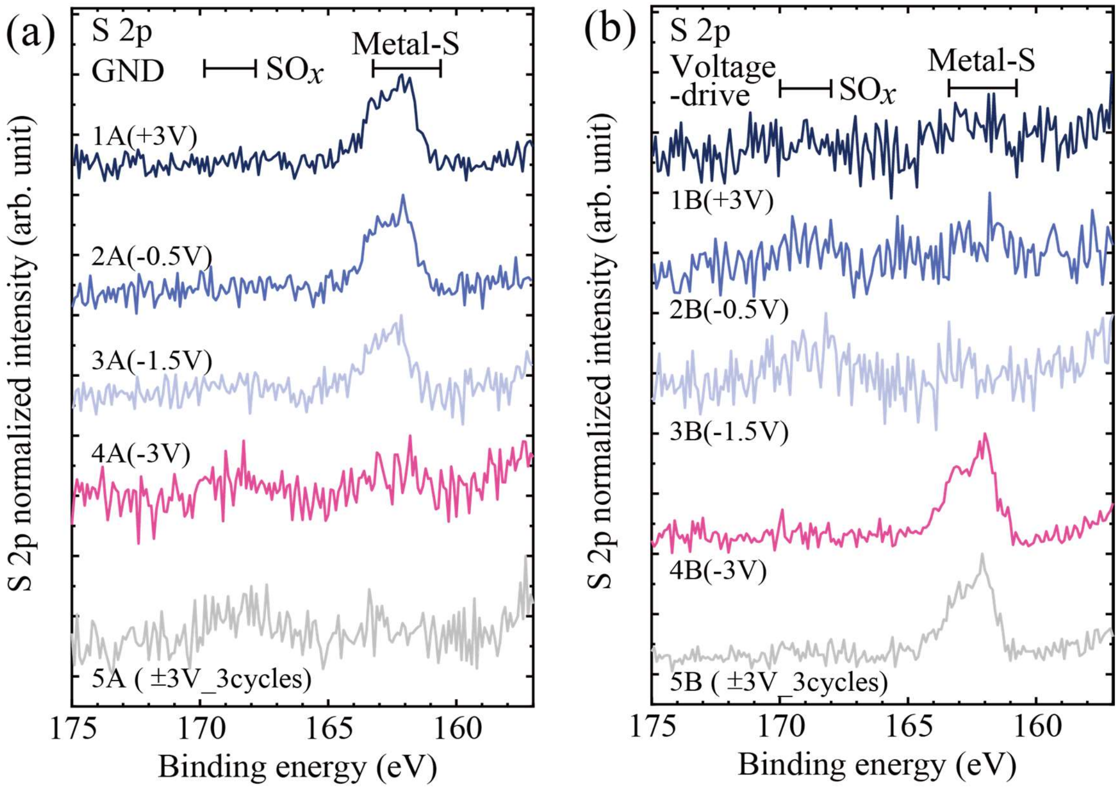

Figure S10. S 2*p* XPS spectra of the reaction sites at the (a) grounded and (b) voltage-drive electrodes.

Figure S11 is the Cu 2*p* 3/2 XPS spectrum measured using the control sample (black line). For the control sample, IL droplet on the reaction sits was washed out without applying external voltage. For comparison, the XPS spectra for the reaction sites 1A and 1B in device X1 are also plotted in Figure S9.

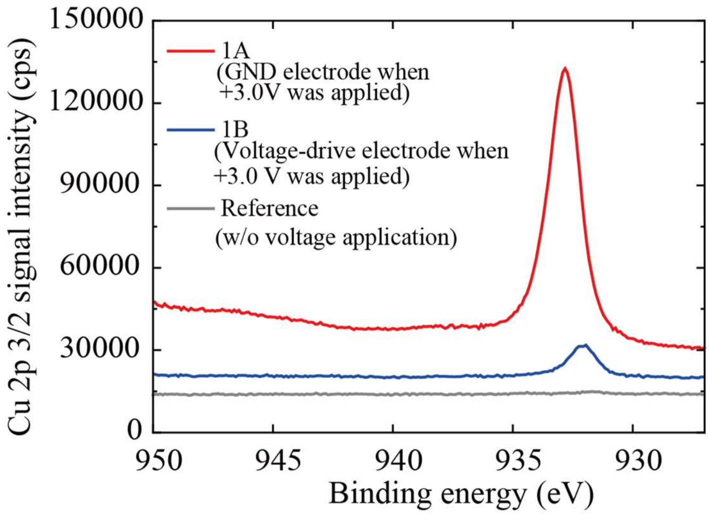

Figure S11. Cu 2*p* 3/2 XPS spectrum measured using the control sample, to which no external voltage was applied. For comparison, Cu 2*p* 3/2 XPS spectra for 1A and 1B are also shown.

Figures S12(a) and S12(b) are the examples of the waveform separation analysis for the Cu 2*p* XPS and Cu LMM Auger spectra. The analyzed sample in these examples corresponds to the reaction site 5A in the device X5 shown in Table 1. As shown in Figure S12(a), the Cu 2*p* XPS spectrum was separated into two component waves labeled as "Cu2*p*_1" and "Cu2*p*_2". The component wave Cu2*p*_1 corresponds to the signal from Cu, Cu_2O, and CuS_x, while the component wave Cu2p_2 corresponds to the signal from $Cu(OH)_2$ etc. Also, as shown in Figure S12(b), the Cu LMM Auger spectrum was separated into two component waves labeled as "CuLMM_1" and "CuLMM_2". The component wave CuLMM_1 corresponds to the signal from Cu, while the component wave CuLMM2 corresponds to the signal from $Cu(OH)_2$ Cu_2O, and CuS_x etc. In Table S1, the areal intensities of the above mentioned component waves together with the amounts of Cu, O, and S detected from each reaction sites in Table 1 are summarized. The Cu compound ratio was calculated using these values.

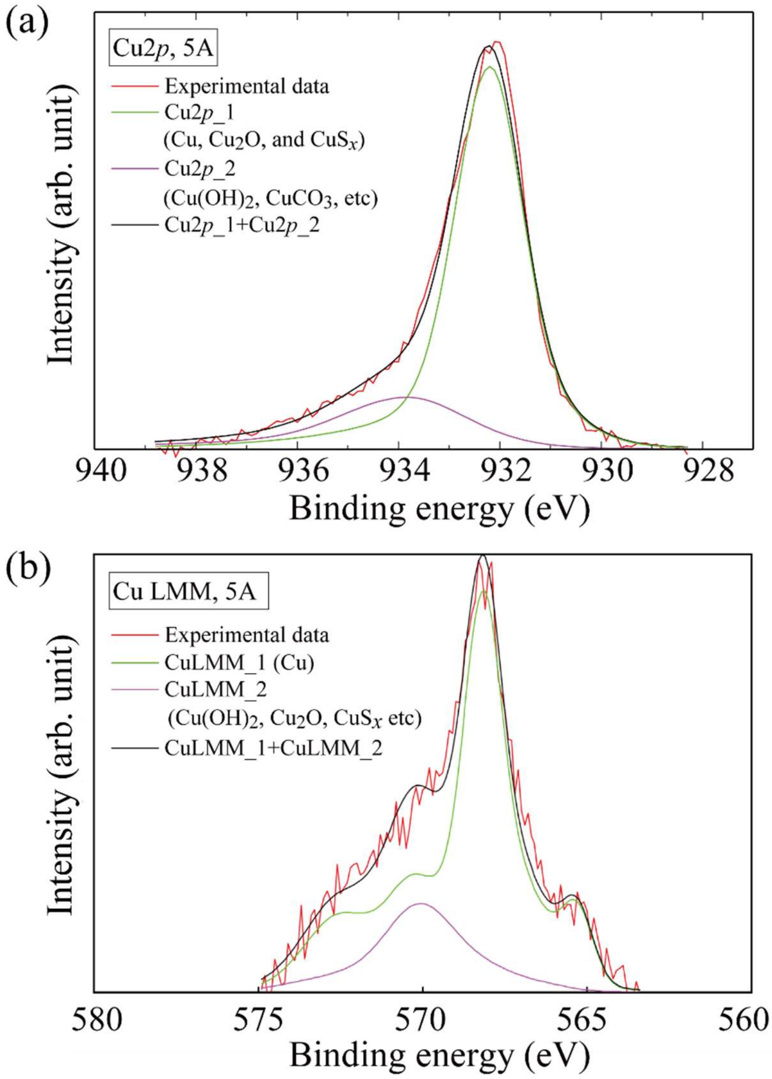

Figure S12. Examples of the waveform separation analysis for (a) the Cu 2*p* XPS and (b) Cu LMM Auger spectra.

Table S1. The areal intensities of component waves (Cu2*p*_1, Cu2*p*_2, CuLMM_1, and CuLMM_2) and Cu, O, and S amounts for each reaction site. Since the lower detection limit in the present XPS measurement was 1 at.%, the tiny amount of S smaller than 1 at.% was neglected when calculating the Cu compound concentration.

Reaction site ID	Cu (at.%)	O (at.%)	S (at.%)	Percentage of areal intensity in Cu 2*p* XPS spectra		Percentage of areal intensity in Cu LMM Auger spectra	
				Cu2*p*_1 (%)	Cu2*p*_2 (%)	CuLMM_1 (%)	CuLMM_2 (%)
1A	36.9	25.7	3.1	100	0	19.42	80.58
1B	3.7	47.0	0.4	92.12	7.88	92.09	7.91
2A	34.1	27.3	2.1	100	0	19.28	80.72
2B	6.1	46.9	0.3	92.10	7.90	91.18	8.82
3A	25.8	35.0	1.9	83.38	16.62	21.09	78.91
3B	7.4	45.6	0.5	81.60	18.40	81.56	18.44
4A	8.6	45.6	0.5	60.77	33.21	60.06	39.94
4B	32.1	30.4	3.8	100	0	34.64	65.36
5A	8.8	44.7	0.6	79.43	20.57	79.24	20.76
5B	43.6	24.4	3.2	100	0	44.89	55.11

Figures S13(a) and S13(b) are the color-coded *I-V* curves, which were used to evaluate STM performance when PH = 1.0 V and 2.5 V, respectively. Here, color coding was conducted based on the three-digits number in the input signal (random stream of 0 and 1), i.e., 000, 100, 010, 110, 001, 101, 011, and 111. All the current data at the time step *T* can be categorized to one of these eight classes.
To improve the visualization of the class separation, we calculated the averaged current-voltage curves for each class, which were plotted in Figures S13(c) and S13(d) together with the class names. These *I-V* curves in Figures S13 (c) and S13 (d) can be regarded as ideal *I-V* curves without cycle-to-cycle variations of the current values. Also, the number on the extreme left, e.g., "0" in "011", corresponds to the target for the STM task when T_{delay} = 2. Therefore, we can evaluate the ideal performance for the STM task with T_{delay} = 2 using the averaged data in Figures S13 (c) and S13 (d) instead of the raw current data in Figures S13 (a) and S13 (b).

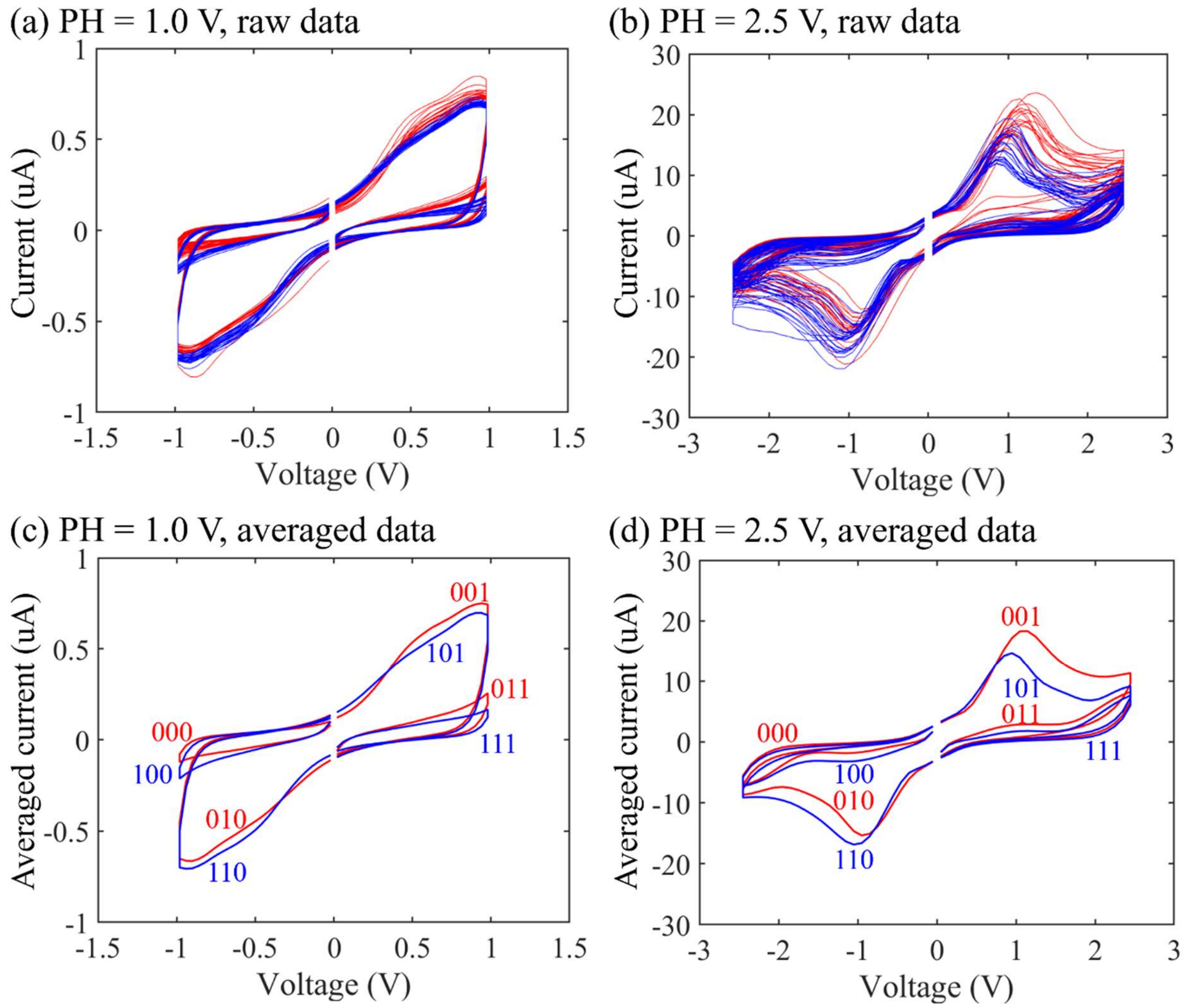

Figure S13. Color-coded raw current data as function of the applied volage for (a) PH = 1.0 V and (b) PH = 2.5 V. Color corded averaged current data for (c) PH = 1.0 V and (d) PH = 2.5 V. The color-coding was conducted based on the three-digits number in the input signal (000, 100, 010, 110, 001, 101, 011, and 111), which are also depicted in (c) and (d).

The STM performances for T_{delay} = 1 to 3 were evaluated using the averaged data and the values of MC as a function of the pulse height (PH) were plotted in Figure S14. Different from the PH dependence of MC evaluated using the raw current values, MC increased with increasing PH when the averaged current waveforms were used for the STM task. This result indicates that the cycle-to-cycle variation of the current value included in the raw current data decreases the STM task performance.

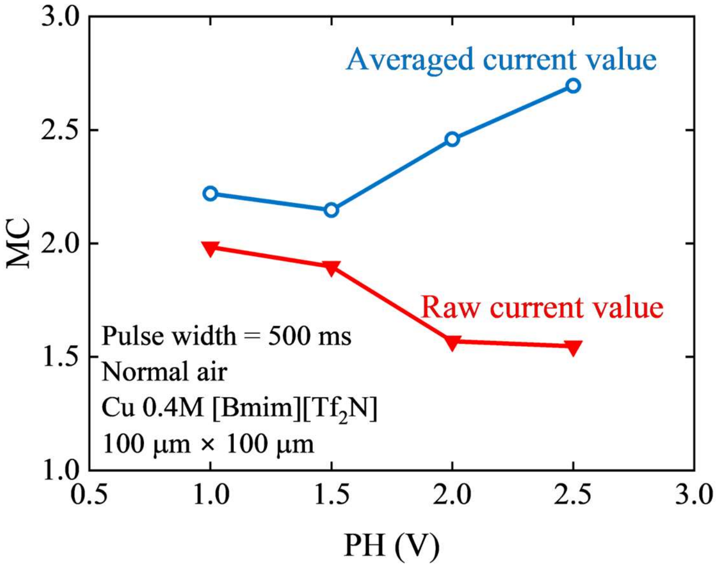

Figure S14. PH dependence of MC in the STM task evaluated using the raw current data and averaged current data.

Figure S15 is the NARMA2 task performance in the evaluation phase using the 100 datasets for training and 298 datasets for evaluation. The value of PH applied to IL-PRD was 2.0 V. The values of CC and NMSE was almost comparable to those evaluated using the 100 datasets for training and 98 datasets for evaluation shown in Figure 10.

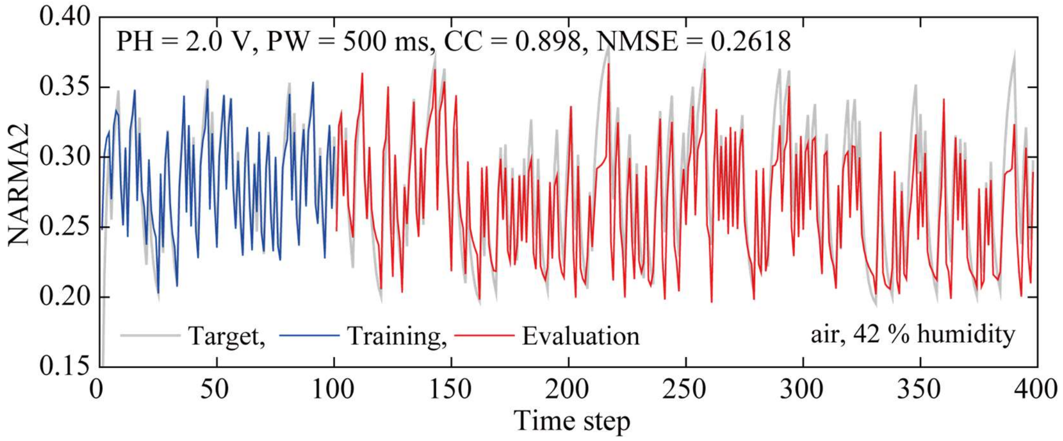

Figure S15. NARMA2 task performance in the evaluation phase using the 100 datasets for training and 298 datasets for evaluation.

Figure S16 shows the influence of the training data number ($N_{training}$) on the correlation coefficient (CC) for the evaluation phase of the NARMA2 task. With increasing $N_{training}$ from 10 to 100, the value of CC saturated. Especially when $N_{training} > 60$, CC was almost independent on $N_{training}$.

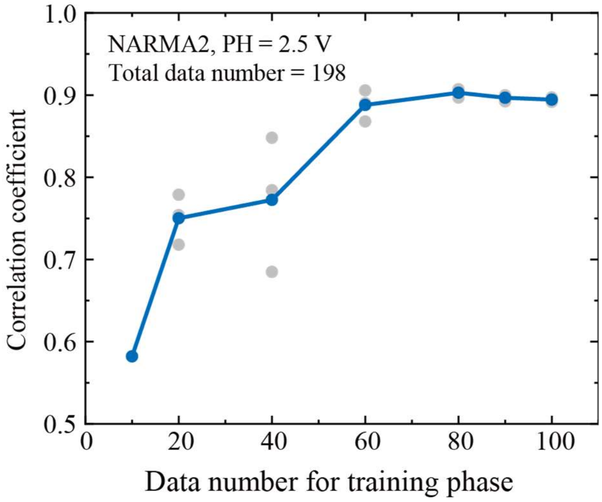

Figure S16. Training data number dependence of correlation coefficient (CC) for the evaluation phase of the NARMA2 task. For each $N_{training}$ value, CC was evaluated 3 times (gray solid circles) and average value was calculated (blue solid circles).

Figures S17 show the virtual node number k dependence of the NARMA2 task performance. The value of k was varied from 5 to 50. The number of datasets for the training and evaluation phases were 100 and 98, respectively. Although NARMA2 task performance monotonically decreases with decreasing the value of k, the value of CC for PH = 2.5 V and k = 25 was almost the same as that for PH = 1.0 V and k = 50.

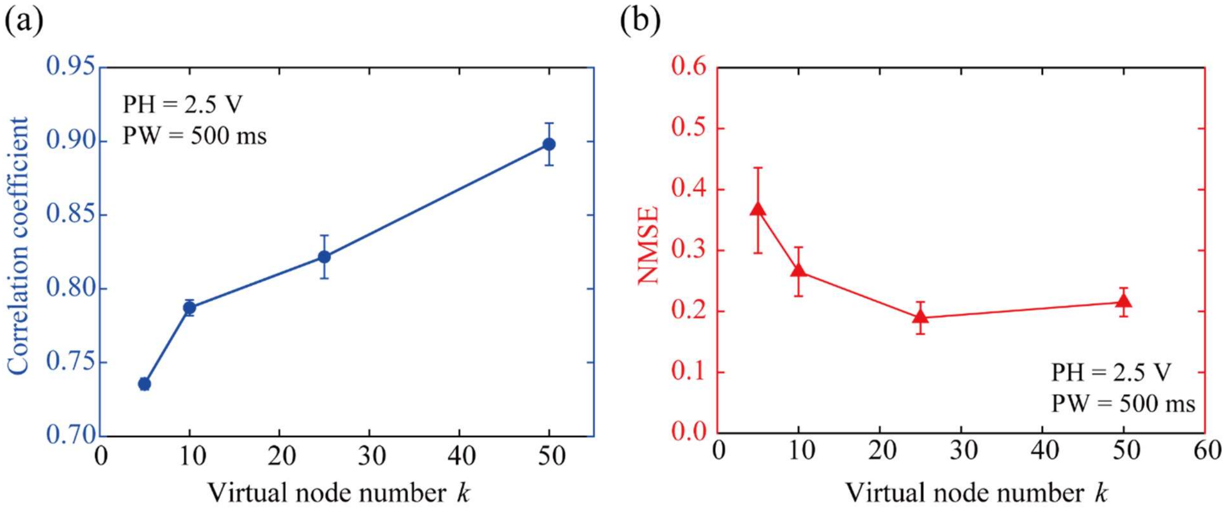

Figure S17. Virtual node number dependence of (a) correlation coefficient and (b) NMSE for the NARMA2 task. The PH and PW of TVPs to acquire the output current dataset from IL-PRD were 2.5 V and 500 ms, respectively.

As shown in Figure S18, the NARMA2 task performance became quite low when the output current from a resistor and linear regression were used. However, by using the deep neural network such as long-short-term memory (LSTM) network, the NARMA2 task performance was improved even when the output current from a resistor was used. Figure S19 is the NARMA2 task performance when the output current from a resistor and LSTM network were used. The layer structure of the network is, input layer, LSTM layer having 10 LSTM units, full-connected layer, drop-out layer, full-connected layer, output layer. By using the LSTM network, the NARMA2 task performance can be improved even when the output current signals without non-linear transformation were fed to the neural network, as shown in Figure S19. The values of CC and NMSE for Figure S19 was 0.97 and 0.077, respectively.

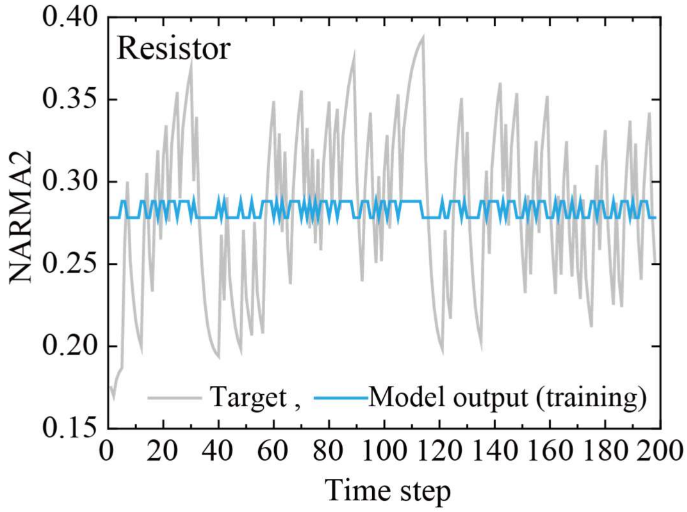

Figure S18. NARMA2 task performance when the output current from a resistor and linear regression were used.

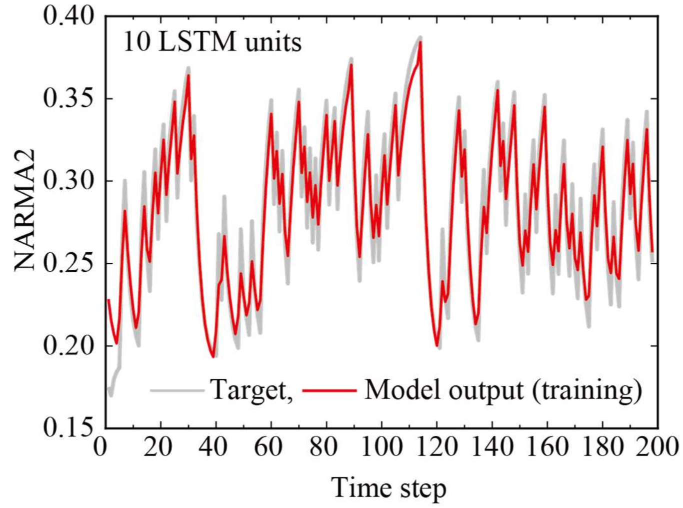

Figure S19. NARMA2 task performance when the output current from a resistor and long-short-term memory (LSTM) network were used.

Figures S20 is the possible array structure of IL-PRD, which has multi electrodes to generate simultaneously a number of output signals to map the input signal to the high-dimensional space. Because each output electrode acts as an actual reservoir node, it is expected that the richness of the reservoir state is improved compared with the isolated IL-PRD.

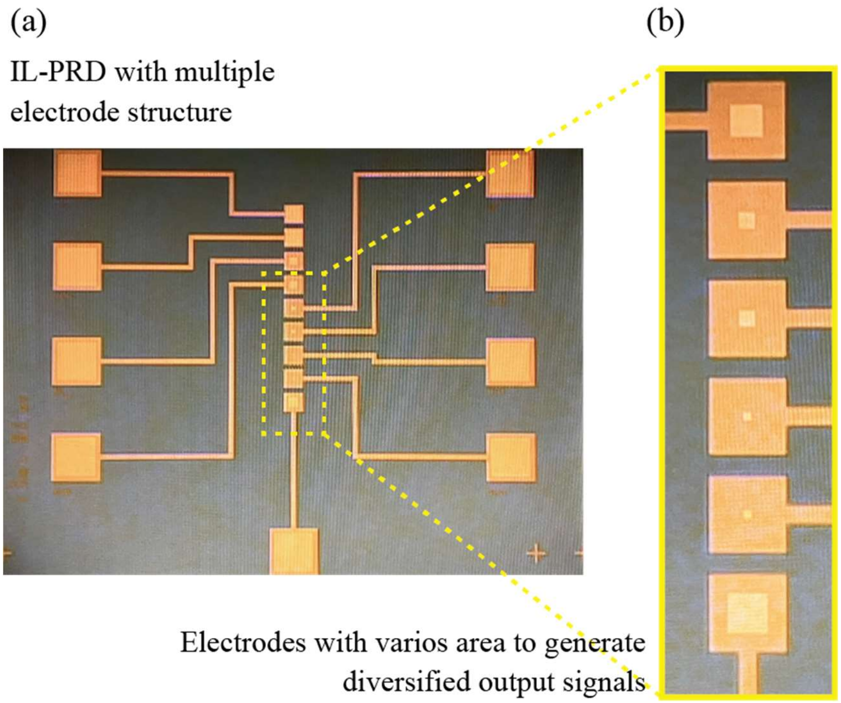

Figure S20. (a) IL-PRD with multiple electrodes to increase the richness of the reservoir state and (b) a part of electrode array having different electrode size to generate diversified output signals.

In Figures S21, we evaluated the influence of the solution resistance by measuring the *I-V* curves for different value of the electrode distance *d* between the input and output electrodes. Each reaction sites in the IL-PRD shown in Figures S21 have the identical area of 100 μm × 100 μm. The value of *d* in Figure S21(a) and S21(b) is 150 μm and 650 μm, respectively. The *I-V* curves for *d* = 150, 400, and 650 μm are shown in Figure 22, which was measured by applying the triangular voltage pulse having the pulse width of 300 ms. Even when the value of *d* became several times as large as that in Figure S21(a), the change in the *I-V* curves is negligibly small, which indicates that the present IL-PRD has a robustness against the change in the solution resistance. Therefore, it is reasonably considered that the variation in the IL droplet thickness and consequent variation in the solution resistance have a negligibly small impact on the electrical property of the present IL-PRD.

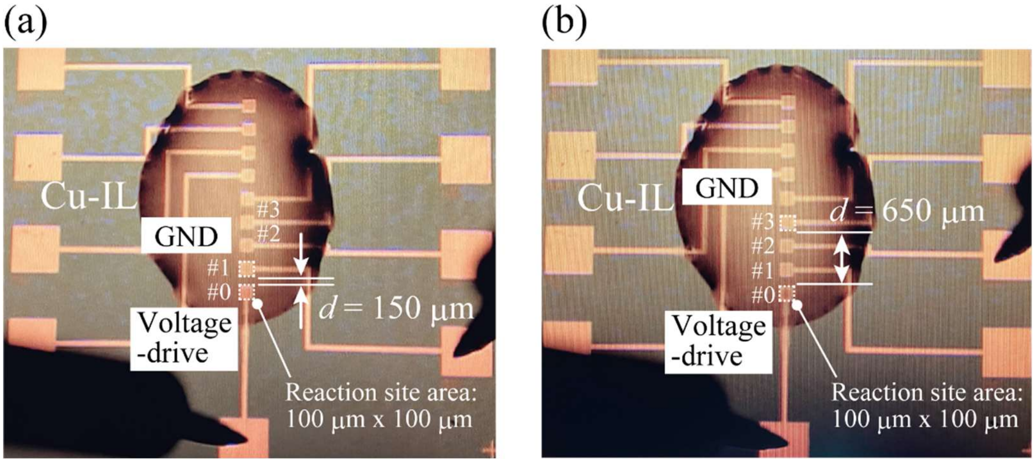

Figure S21. The measurement configurations to evaluate the influence of the solution resistance. All the reaction site area in the present IL-PRD is 100 μm × 100 μm. The electrode distance between the input and output electrode is (a) 150 μm and (b) 650 μm. The electrodes surrounded by the white dotted lines were used to measure the *I-V* curves. #0 ~ #3 are the identification number of the electrodes.

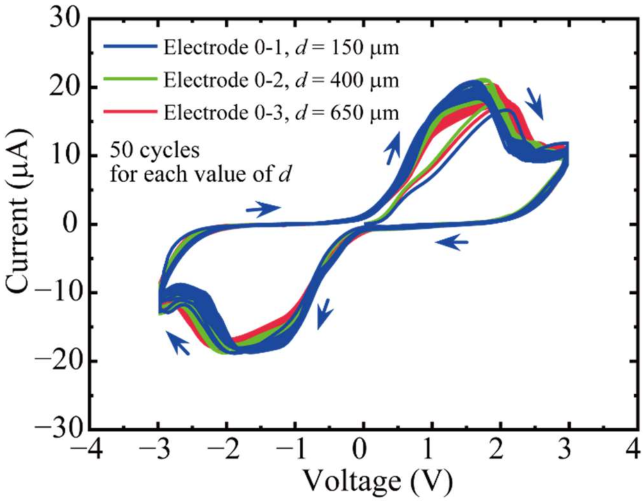

Figure S22. The electrode distance *d* dependence of the *I-V* curves in IL-PRD with *d* = 150, 400, and 650 μm. The blue arrows indicate the direction of the voltage change.

Figure S23 is an example of the confusion matrix used to calculate the ECG signal classification accuracy when the epoch number was 5. In the present study, the classification accuracy was evaluated using the 300 datasets for the test phase (100 each of the ARR, CHF, and NSR signals). In this case, a part of the ARR and NSR signals was classified wrongly, while all of the CHF signals were classified correctly.

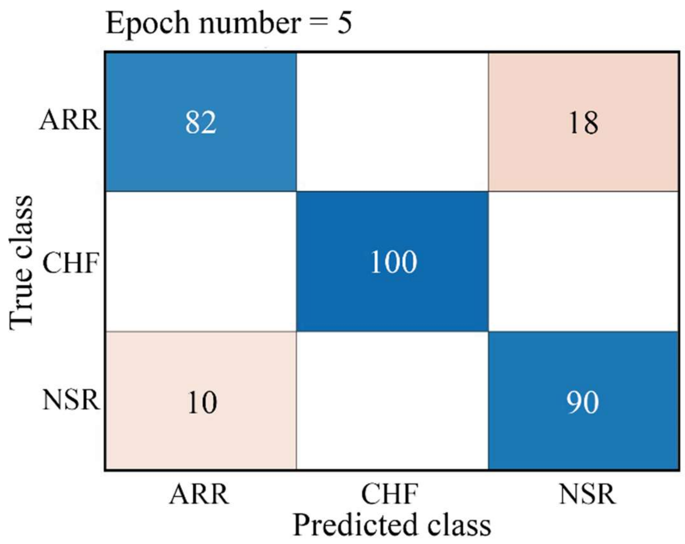

Figure S23. An example of the confusion matrix for the ECG signal classification task.
